# A novel IBA57 variant is associated with mitochondrial iron–sulfur protein deficiency and necrotizing myelopathy in dogs

**DOI:** 10.3389/fgene.2023.1190222

**Published:** 2023-07-12

**Authors:** Paul J. J. Mandigers, Oliver Stehling, Manon Vos-Loohuis, Frank G. Van Steenbeek, Roland Lill, Peter A. Leegwater

**Affiliations:** ^1^ Expertise Centre of Genetics, Department of Clinical Sciences, Faculty of Veterinary Medicine, Utrecht University, Utrecht, Netherlands; ^2^ Institut für Zytobiologie und Zytopathologie and Center for Synthetic Microbiology Synmikro, Philipps-Universität Marburg, Marburg, Germany

**Keywords:** spinal cord, leukodystrophy, Kooikerhondje, Kooiker dog, canine, IBA57-R147W, respiratory complexes, lipoyl synthase

## Abstract

**Introduction:** Hereditary necrotizing myelopathy (HNM) in young Kooiker dogs is characterized by progressive ataxia and paralysis with autosomal recessive inheritance. The basic genetic defect is unknown. We investigated the possible cause by a genome-wide analysis using six affected and 17 unrelated unaffected Kooiker dogs and by functional follow-up studies.

**Method:** The HNM locus was mapped by a case–control study using a dense SNP array and confirmed by linkage analysis of two pedigrees. The gene exons in the critical region were analyzed by next-generation sequencing. The functional effect of the candidate canine *IBA57* pathogenic variant was biochemically examined in an established HeLa cell culture model in which the endogenous *IBA75* gene product was depleted by RNAi.

**Results:** The basic defect was localized in the centromeric 5 Mb region of canine chromosome 14. The most associated SNP co-segregated fully with HNM and reached an LOD score of 6.1. A candidate pathogenic mutation was found in the iron–sulfur cluster assembly gene *IBA57* and led to the amino acid substitution R147W. The expression of human IBA57 harboring the canine R147W exchange could only partially restore the biochemical defects of several mitochondrial [4Fe-4S] proteins upon IBA57 depletion, showing that the mutant protein is functionally impaired.

**Discussion:** Pathogenic variants in human *IBA57* cause multiple mitochondrial dysfunction syndrome 3 (MMDS3), a neurodegenerative disorder with distant similarities to HNM. The incomplete functional complementation of IBA57-depleted human cells by IBA57-R147W identifies the DNA mutation in affected Kooiker dogs as the genetic cause of HNM. Our findings further expand the phenotypic spectrum of pathogenic *IBA57* variants.

## Introduction

A variety of inherited neurological disorders have arisen from spontaneous mutation events in dog breeds. These diseases present models for human diseases with shared molecular defects (van Steenbeek et al., 2016). As most dog breeds tend to be inbred and subjected to artificial selection by the breeders, these spontaneous mutations can reach undesirable frequencies. A notable example is degenerative myelopathy in dogs, caused by mutations in *SOD1*, known for its involvement in amyotrophic lateral sclerosis in humans (Zeng et al., 2014). Less well characterized is the hereditary necrotizing myelopathy (HNM) affecting young dogs of the breed Nederlandse Kooikerhondje (named Kooiker dogs hereafter), which has been described earlier by Mandigers et al. (1993). The clinical signs, paresis and ataxia, start in most dogs around the age of 3–12 months in the hind limbs and progresses to tetraparalysis before the age of 2 years. Post-mortem examination performed in these dogs revealed a symmetric bilateral necrotizing myelopathy with malacia in the ventral and dorsal white matter. Pedigree analysis indicated that the disease is inherited as an autosomal recessive trait, probably originating from a single founder (Mandigers et al., 1993). Here, we present the identification of an associated variant and the functional evaluation of the defect gene product, a variant of *IBA57* encoding a component involved in mitochondrial [4Fe-4S] protein assembly (Lill and Freibert, 2020). According to our findings, the *IBA57* mutation is the likely cause of the observed disease phenotype.

IBA57 is a nuclear-encoded mitochondrial protein functioning together with ISCA1 and ISCA2 in the reductive fusion of [2Fe-2S] clusters to a [4Fe-4S] cofactor (Sheftel et al., 2012; Weiler et al., 2020). The [2Fe-2S] cluster precursor is initially assembled on the ISCU2 scaffold protein involving sulfur delivery from the cysteine desulfurase complex consisting of NFS1, ISD11, and the acyl carrier protein ACP (Webert et al., 2014; Boniecki et al., 2017; Freibert et al., 2021). Subsequently, the [2Fe-2S] cluster is transferred by a HSCA9-HSC20-GRPE chaperone system to glutaredoxin 5 (GLRX5) to serve as a substrate for the ISCA1-ISCA2-IBA57 complex (Kim et al., 2010; Ye et al., 2010; Uzarska et al., 2013). The [4Fe-4S] cluster products are consecutively incorporated into apo-client proteins, a process assisted by several targeting factors such as NFU1 (Sheftel et al., 2009; Cameron et al., 2011; Navarro-Sastre et al., 2011). Defects in the mitochondrial [4Fe-4S] protein assembly impact numerous metabolic pathways, and depending on the affected Fe/S protein, the assembly factors are grouped into one of five types of multiple mitochondrial dysfunction syndromes (MMDS) (Lill and Freibert, 2020; Lebigot et al., 2021; Camponeschi et al., 2022), such as MMDS3 caused by mutations in IBA57 (OMIM 615330) (Ajit Bolar et al., 2013; Debray et al., 2015; Lossos et al., 2015). Irrespective of the genetic basis, MMDS types 1–5 are characterized by insufficient lipoyl cofactor synthesis, tricarboxylic acid cycle, and respiratory chain function and associated with malfunctions of the central nervous system. Our genetic and biochemical analyses suggest that the identified *IBA57* mutation is causative for the MMDS3-related mitochondrial deteriorations, eliciting the HNM phenotype in Kooiker dogs.

## Materials and methods

### Dogs

Young Kooiker dogs born in Europe, which displayed neurological signs suggestive of myelopathy, were referred for clinical examination to the first author (PM) at the Department of Clinical Sciences of the Faculty of Veterinary Medicine of Utrecht University (Table 1). If the neurological signs were conclusive for myelopathy and the clinical features were in agreement with HNM (Mandigers et al., 1993), additional diagnostics was limited to a routine blood examination, a toxoplasmosis titer, and on request of the owner, an MRI scan, under generalized anesthesia, of the cervicothoracic spinal cord. For the generalized anesthesia, the dogs were premedicated 15 min prior to induction with atropin at a dose of 0.03 mg/kg intramuscularly. The dogs were induced with 0.5 mg/kg of methadone (methadone, Pharmacy Department, Utrecht University) intravenously and 0.5 mg/kg of midazolam (midazolam in Dormicum Roche Netherlands BV, Mijdrecht) intramuscularly. The dogs were further anesthetized using propofol (propofol, Fresenius Kabi, Netherlands BV, Den Bosch) (based on an effect of approximately 2 mg/kg) and, after intubation, maintained on 0.5 L of oxygen, 1 L of air, and approximately 1% of isoflurane (Abbott Animal Health). The dogs remained under the clinical supervision of the first author, and at deterioration of neurological signs, an elective euthanasia, using 5 mL of EUTHASOL^®^ intravenously (AST Pharma BV, Oudewater, Netherlands), was performed. For post-mortem examination, the brain and spinal cord were fixed in 4% buffered formaldehyde and examined to confirm the diagnosis of HNM as described earlier (Mandigers et al., 1993).

**TABLE 1:** List of Kooiker dogs affected by hereditary necrotizing myelopathy.

Dog	Name	Gender	Birthdate	Country
1	Max	Male	05-04-2001	The Netherlands
2	Thora	Female	10-08-2006	The Netherlands
3	Abby	Male	16-12-2009	Germany
4	Celina	Female	01-04-2010	Germany
5	Curty	Male	20-05-2010	Germany
6	Carino	Male	30-04-2009	Germany
7	King	Male	25-02-2012	The Netherlands
8	Sowiedu	Male	02-11-2012	The Netherlands

DNA samples were extracted from EDTA-blood of the dogs using a chemagic™ MSM I robot (Perkin Elmer). The samples of unaffected, related, and unrelated Kooiker dogs were retrieved from our DNA bank containing approximately 2,800 Kooiker dog samples, stemming from research and DNA diagnostics for von Willebrand disease in the breed (van Oost et al., 2004). Again, all these samples were extracted from EDTA-blood. All dogs were privately owned and included in the study with the informed consent of their owners. Thus, we complied with the conditions set forth in the Dutch ‘Wet op de Uitoefening van de Diergeneeskunde’ (Law on the Practice of Veterinary Medicine) of 21 March 1990, and the approval by an animal ethics committee for the use of samples was not necessary.

### Gene mapping

\The DNA samples of six Kooiker dogs with HNM (Table 1) and 17 unrelated, unaffected Kooiker dogs that were older than 1 year and clinically unaffected were genotyped using the Illumina CanineHD SNP array. The DNA samples and arrays were processed at the Centre National de Génotypage, Paris, France, at the time in the frame of the LUPA European project^1^. A case–control comparison of allele frequencies was performed with the PLINK software version 1.07.

The SNP BICF2G630517911 was genotyped in available samples from two pedigrees that segregated HNM (Supplementary Figure S1). The oligonucleotides for PCR amplification and chain termination DNA sequence analysis, performed as outlined in the following section, are included in Supplementary Table S1. The LOD score for linkage was calculated with a stand-alone version of the SuperLink version 1.7 (Fishelson and Geiger, 2004).

### DNA sequence analysis

One affected Kooiker dog and two unaffected control dogs were selected for targeted resequencing of genomic DNA. After DNA was sheared, fragments of all exons and adjacent intronic 35 bp of genes from the critical region were enriched for and amplified using an Agilent SureSelect in-solution enrichment design. Library preparation was performed using the Illumina TruSeq^®^ Nano DNA Prep Kit. The enriched libraries were sequenced on an Illumina MiSeq. Mapping and variant calling of the paired-end reads of 150 bases were achieved using a custom bioinformatic pipeline based on the Burrows–Wheeler Aligner algorithm (Li and Durbin, 2009). The SAMtools was used for annotation and initial variant calling against the reference genome CanFam3.1 (Li et al., 2009). The variants were curated by comparing the allele frequencies of detected variants in a cohort of 590 dogs (Supplementary Table S2). Two variants of interest were further analyzed in all available dogs of the HNM pedigrees by chain termination sequencing using the BigDye v3.1 (Applied Biosystems) on a Genetic Analyzer 3500xL (Applied Biosystems). The oligonucleotides used for PCR amplification and sequencing reactions are specified in Supplementary Table S1.

### Tissue culture–based IBA57 complementation assays and plasmid generation

HeLa cells were cultured at standard conditions and transfected thrice in a 3-day interval by electroporation as described by Stehling et al. (2009), Sheftel et al. (2012), and Ajit Bolar et al. (2013), allowing for RNAi-mediated depletion and concomitant plasmid-based expression of silently mutated, RNAi-resistant, wild-type IBA57 (smIBA57^wt^). The smIBA57^wt^-encoding plasmid (Debray et al., 2015) used in this procedure further served as a template to introduce the Kooiker dog p.Arg147Trp (R147W)–encoding genomic mutation into the human IBA57 open reading frame. The resulting plasmid smIBA57^R147W^ was used to study the HNM-related mutation in the complementation assays. Plasmid modification was achieved using the Q5^®^ Site-Directed Mutagenesis Kit (New England Biolabs) according to the protocol of the manufacturer. The mutagenesis primer sequence was TCCGGTGGAAGGTCAC, with the mutant base underscored, and the reverse primer sequence was TCCTGTATAGCGCGA. The DNA sequence of the complete IBA57 coding region of the mutant plasmid was verified by dideoxy DNA sequencing. For subsequent biochemical analyses, the transfected HeLa cells were harvested by trypsination, and sample aliquots were subjected either to immunoblotting or digitonin-based cell fractionation, followed by spectrophotometric determination of enzyme activities as published (Biederbick et al., 2006; Stehling et al., 2009; Stehling et al., 2018b).

### Antibodies

IBA57, NFU1, and CIAO3 were detected by polyclonal antisera raised in rabbits against recombinant human full-length proteins purified from *E. coli* (Navarro-Sastre et al., 2011; Sheftel et al., 2012; Stehling et al., 2018a). Purified polyclonal rabbit antibodies directed against UQCRFS1, UQCRC2, COX2, COX6A/B, and F1α/β were a kind gift from H. Schägger and I. Wittig (Frankfurt, Germany). Purified rabbit anti-GPAT was kindly provided by H. Puccio (Illkirch, France), and mouse anti-IRP1 was a kind donation from R. Eisenstein (Madison, United States). Mouse monoclonal antibodies directed against NDUFS1, NDUFS8, NDUFV2, NTHL1, or β-actin, as well as rabbit anti-DPYD, were availed from Santa Cruz Biotechnology. Mouse monoclonal antibodies directed against NDUFA9, NDUFA13, NDUFB4, NDUFB6, or SDHB were obtained from MitoSciences via Abcam. Mouse anti-DLAT (Cell Signaling Technology, 4A4-B6-C10), anti-GAPDH (Calbiochem), and anti–α-tubulin (clone DM1a, Sigma Aldrich) and rabbit anti-ACO1 (Invitrogen), anti-lipoic acid (Merck/Calbiochem), or anti-POLD1 (PTG Lab) were also commercially available.

### Statistical analysis

Data are presented as mean ± SD and analyzed by one-way repeated measures ANOVA, followed by Dunnett’s *post hoc* test. The smIBA57^wt^ data served as the control in order to individually determine the significance levels of IBA57 depletion phenotypes as well as of smIBA57^wt^ and smIBA57^R147W^ complementation phenotypes. *p < 0.05, **p < 0.01, and ***p < 0.001.

## Results

### Dogs and clinical findings

Eight young Kooiker dogs with clinical signs of HNM were screened between 2001 and 2014 (Table 1). All eight dogs (six males and two females) had a history of progressive hind limb paresis and ataxia with an onset between 4 and 12 months of age. Neurological examination revealed postural deficits and exaggerated spinal reflexes in the hind limbs in all eight dogs. Two dogs also showed ataxia of the front limbs and postural deficits. Based on the neurological examination, the neuro-localization was in the cervicothoracic/thoracolumbar region. As the clinical presentations were highly similar to cases observed earlier (Mandigers et al., 1993), the diagnostics was limited to a routine blood examination and toxoplasmosis titer, which revealed no abnormalities in all eight dogs. In one dog, an MRI scan was performed that revealed abnormalities suggestive of spinal cord disorder (Truar et al., 2012). The health condition of all dogs deteriorated within the following months, pressing the owners to elect euthanasia. Post-mortem examination performed in these eight dogs revealed similar pathological lesions as described earlier (Mandigers et al., 1993), revealing a symmetric bilateral necrotizing myelopathy with malacia in the ventral and dorsal white matter, thus confirming the clinical diagnosis.

### Gene mapping and mutation detection

**FIGURE 1:**
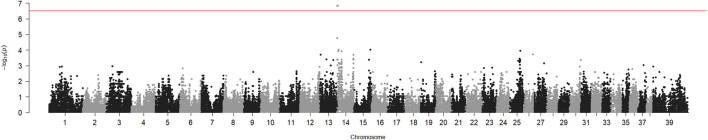
Genome-wide association analysis of hereditary necrotizing myelopathy in Kooiker dogs. Chi-square *p*-values for SNP allele number differences between six cases and 17 unaffected dogs were calculated with PLINK and plotted against the chromosome number and position on the chromosome. Chromosome 39 represents the X chromosome. The peak near the telocentric centromere of chromosome 14 represents the six SNPs, which includes BICF2G630517911.

The genome-wide association analysis of six HNM-affected Kooiker dogs from different litters and 17 unrelated controls from the same breed suggested involvement of the region adjacent to the centromere of chromosome 14 (CFA14). The *p*-value of the chi-square test for this region reached 1.7 10^−7^ (Figure 1). The SNP allele frequencies in the region showed that the cases were identically homozygous from the beginning of the chromosome to position 4719754 (SNP BICF2G630519065, CanFam3.1) on CFA14. The segregation of SNP BICF2G630517911 on position 3021087 was analyzed in two extended pedigrees, which included two more patients in addition to the six cases that were included in the genome-wide association analysis (Supplementary Figure S1). The allele of this SNP shared by the cases had a frequency of 0.14 in the controls of the genome-wide analysis. The LOD score for linkage between HNM and the SNP in the two pedigrees was 6.1 without recombinations.

The critical region contained 38 genes (Supplementary Table S3). The exons and bordering DNA sequences of these genes from one case and two controls were enriched and analyzed by next-generation sequencing techniques. One variant in *IBA57* and one in *OBSCN* remained the causative candidates for HNM after quality control and filtering of all observed variants.

The observed variant in *OBSCN* changed an arginine codon to a stop codon.

The annotation was CFA14:g.693260G>A, resulting in XM_038686046.1:c.17228C>T and XP_038541974.1:p.Arg5690*, truncating the largest isoform of obscurin by 3,325 amino acids. Obscurin is a large cytoskeletal protein in the striated muscles, and variants have been associated with cardiomyopathies in humans, although the relevance of the latter has been questioned (Grogan and Kontrogianni-Konstantopoulos, 2019; Fukuzawa et al., 2021). The available DNA samples of dogs from the extended pedigrees were genotyped for the two candidate variants, and both co-segregated with HNM. However, the two variants were not in complete linkage disequilibrium. Of the 136 heterozygous carriers of the *IBA57* variant that were detected in our DNA bank, three dogs did not carry the *OBSCN* mutation. A total of 1,522 Kooiker dogs were analyzed, yielding an allele frequency of 0.04, which is not unusual in dog genetics.

**FIGURE 2:**
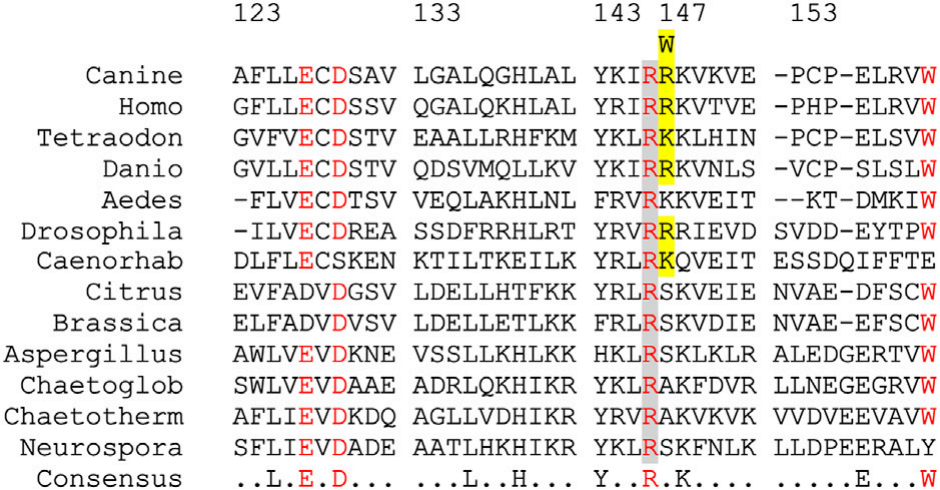
Multi-sequence alignment of the IBA57 segment carrying the canine R147W amino acid exchange. Partial IBA57 amino acid sequences from the indicated organisms were aligned using Multalin (Corpet, 1988). The partial conservation of canine residue R147 in IBA57 is highlighted in yellow, and the strict conservation of the preceding R146 is in gray. Homo, *Homo sapiens*; Tetraodon, *Tetraodon nigroviridis*; Danio, *Danio rerio*; Aedes, *Aedes aegypti*; *Drosophila*, *Drosophila melanogaster*; Caenorhab, *Caenorhabditis elegans*; Citrus, *Citrus sinensis*; Brassica, *Brassica carinata*; Aspergillus, *Aspergillus fumigatus*; Chaetoglobo, *Chaetomium globosum*; Chaetotherm, *Chaetomium thermophilum*; Neurospora, *Neurospora crassa*.

The *IBA57* variant annotation was CFA14:g.801179G>A, corresponding to XM_038686047.1:c.439C>T and XP_038541975.1:p.Arg147Trp. The mutated arginine residue is part of a short loop between β-strand 6 and α-helix 2 of the protein that is conserved in animals but not in, e.g., plants and fungi (Figure 2) and also not in bacterial YgfZ relatives (Waller et al., 2010; Weiler et al., 2020).

### IBA57 complementation assay

**FIGURE 3:**
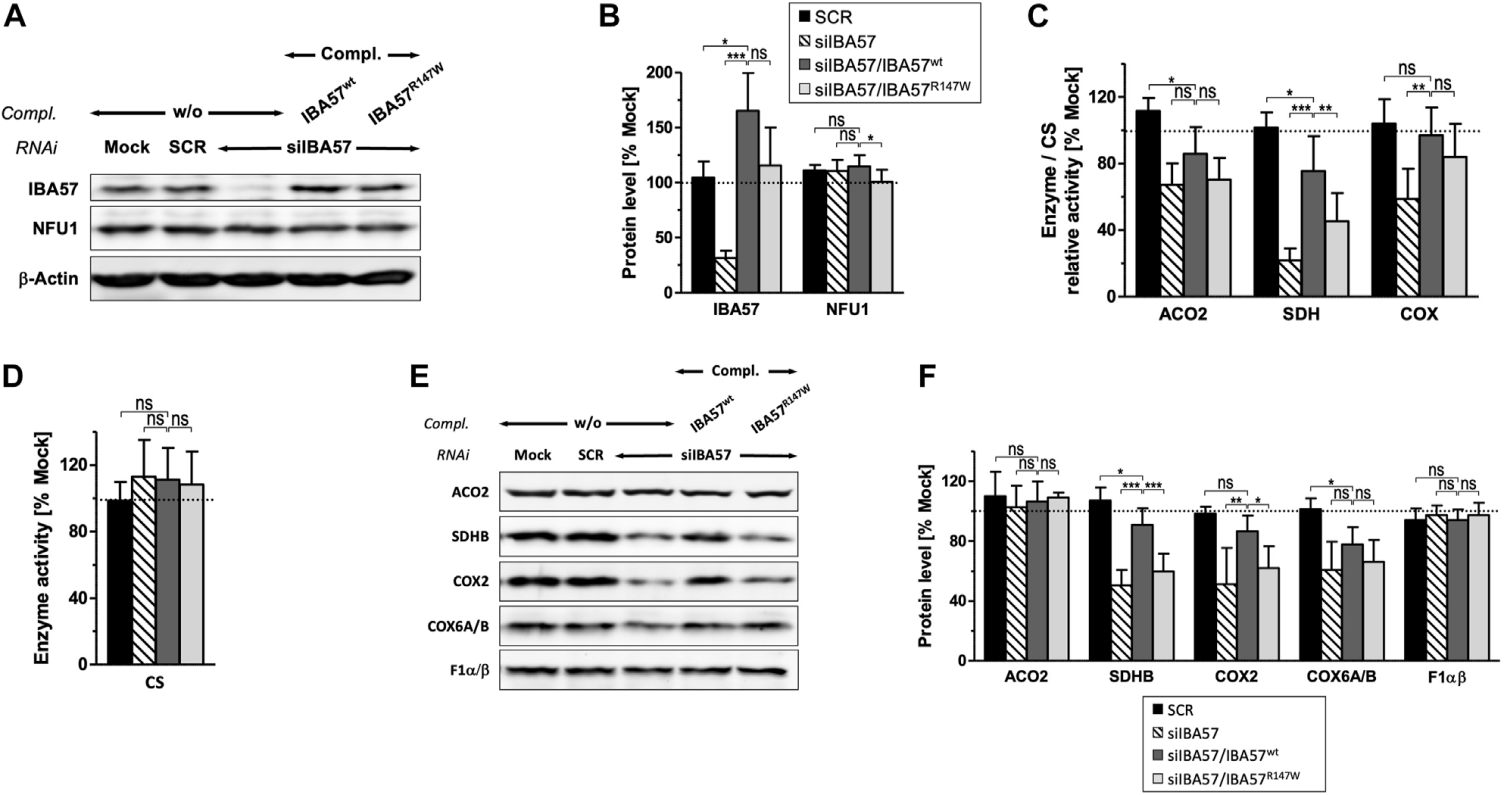
IBA57-R147W amino acid exchange compromises mitochondrial [4Fe–4S] proteins. HeLa cells were transfected thrice at a 3-day interval with scrambled control siRNAs (SCR) or with an IBA57-directed siRNA (siIBA57) in combination with complementing (compl.) plasmids encoding RNAi-resistant wild-type or HNM-related IBA57 (smIBA57^wt^ or smIBA57^R147W^, respectively). As a control, cells were mock-transfected. Analyses were performed with cells harvested after the third round of transfection (9 days of IBA57 depletion). **(A)** Total cell lysates were subjected to immunoblotting and analyzed for the protein levels of the indicated ISC proteins and β-actin as a loading control. **(B)** Immunoblot signals from part **(A)** were quantified relative to β-actin levels, and the ratio was normalized to mock-transfected control cells (dashed line). **(C, D)** Specific activities of mitochondrial aconitase (ACO2), SDH (RCC-II), and COX (RCC-IV) **(C)** were determined in mitochondria-containing membrane fractions prepared by digitonin-based cell fractionation, related to citrate synthase (CS) activity **(D)**, and normalized to mock-transfected control cells. **(E)** Total cell lysates were subjected to immunoblotting as in **(A)** and analyzed for the steady-state levels of mitochondrial proteins and RCC subunits as indicated. **(F)** Immunoblot signals from **(E)** were quantified, and the protein per β-actin ratios (see A) were normalized to mock-transfected control cells. Representative blots are shown. All values are given as the mean ± SD (n = 3–4); *p < 0.05; **p < 0.01; ***p < 0.001; ns, not significant; dashed lines, 100% value of mock-transfected control cells.

To test whether the IBA57-R147W amino acid exchange causes a mitochondrial phenotype and thus relates the Kooiker dog HNM to the MMDS3 spectrum in humans, we applied a well-established HeLa tissue culture complementation approach (Ajit Bolar et al., 2013; Debray et al., 2015; Lossos et al., 2015). In this assay, endogenous IBA57 was depleted by three consecutive rounds of RNAi, and concomitantly, a siRNA-resistant version of human *IBA57* (s*mIBA57*) harboring the HNM c.439C>T mutation was ectopically expressed from a plasmid. After a total tissue culture time of 9 days, RNAi decreased the protein level of endogenous IBA57 by 60%–80%, whereas the abundance of the ISC factor NFU1 was not altered (Figures 3A, B). Expression of wild-type IBA57 (smIBA57^wt^) or R147W mutant protein (smIBA57^R147W^) restored IBA57 to even higher than the control levels, enabling the analysis of mutant smIBA57^R147W^ for its capacity to complement the mitochondrial defects resulting from RNAi-mediated IBA57 depletion. The enzyme activity measurements in IBA57-deficient cells revealed defects in the [4Fe-4S] cluster–containing proteins mitochondrial aconitase (ACO2) and succinate dehydrogenase (SDH) by more than 30% and 75%, respectively, but not in the Fe/S cluster–devoid reference enzyme citrate synthase (Figures 3C, D). Particularly, the decrease in SDH activity was so severe that it also elicited a decline in the steady-state level of the Fe/S cluster–binding subunit SDHB (Figures 3E, F), which is in line with previously observed instability of Fe/S proteins in their apo-form (Stehling et al., 2018b).

The expression of smIBA57^wt^ could recover both ACO2 and SDH enzyme activities as well as the amount of SDHB up to the control levels (Figures 3C, F). By contrast, the Kooiker dog HNM mutation–containing protein smIBA57^R147W^ was inefficient in restoring normal mitochondrial [4Fe-4S] protein assembly. The mutant protein elicited no improvement of ACO2 activity and corrected SDH activity to not more than 50% of wild-type smIBA57.

**FIGURE 4:**
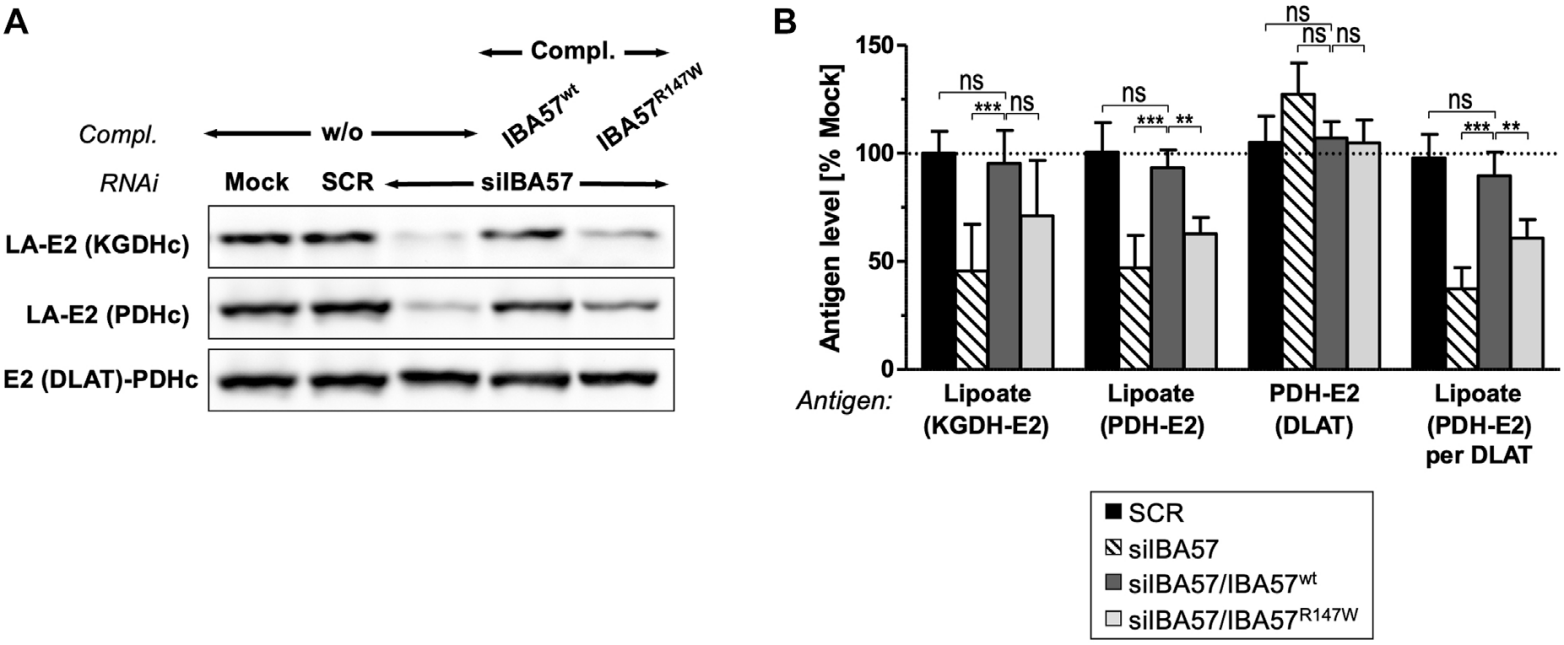
IBA57-R147W amino acid exchange compromises lipoyl cofactor formation. HeLa cells were depleted for IBA57 and treated as in Figure 3A. LIAS activity was indirectly determined by immunoblotting for lipoylation (LA) of KGDHc-E2 and PDHc-E2. DLAT (PDHc-E2 protein) was immunostained for comparison. **(B)** Immunoblot signals from **(A)** were quantified, and the antigen per β-actin ratio was normalized to mock-transfected control cells (dashed line). Representative blots are shown. All values are given as the mean ± SD (n = 3-4); *p < 0.05; **p < 0.01; ***p < 0.001; ns, not significant.

IBA57 deficiency compromises TCA cycle function not only directly via impaired aconitase and SDH function but also indirectly by impairing protein lipoylation catalyzed by the mitochondrial [4Fe-4S] protein lipoic acid synthase (LIAS) (Sheftel et al., 2012; Debray et al., 2015). Probing for the lipoyl cofactors bound to the E2 subunits of the alpha-ketoglutarate dehydrogenase complex (KGDHc) and of the pyruvate dehydrogenase complex (PDHc) by immunoblotting revealed a severe defect in IBA57-deficient cells that was restored to the reference levels by smIBA57^wt^ but was only insufficiently complemented by the smIBA57^R147W^ mutant protein (Figures 4A, B). By contrast, the levels of PDH-E2 (DLAT) protein were neither altered by RNAi nor by complementation. Together, these results indicate a limited LIAS maturation activity in the IBA57-R147W mutant protein–expressing cell.

Similar to SDHB, other Fe/S cluster–containing respiratory chain complex (RCC) subunits are also less stable without their cofactors (Sheftel et al., 2012; Torraco et al., 2017). Accordingly, protein levels of the two RCC-I [4Fe-4S] subunits NDUFS1 and NDUFS8 were decreased upon RNAi-mediated IBA57 deficiency (Supplementary Figure S2A, B). Since both NDUFS1 and NDUFS8 join the N and Q module assembly intermediates of RCC-I at early assembly steps (Guerrero-Castillo et al., 2017), their decay impaired RCC-I formation in general and resulted in the destabilization of other sensitive protomers, which includes the [2Fe-2S] subunit NDUFV2 and non-Fe/S subunits NDUFA13 and NDUFB4, whereas more stable subunits like NDUFA9 and NDUFB6 remained unaffected (Supplementary Figure S2A–D), which is in line with previous observations (Sheftel et al., 2009; Sheftel et al., 2012). Complementation of RNAi-mediated IBA57 deficiency by wild-type smIBA57 restored the majority of tested RCC-I subunits to the control levels, whereas the IBA57-R147W mutant protein only partially recovered NDUFS1 and NDUFS8 and entirely failed to recover the other sensitive RCC-I subunits. This difference in complementation between wild-type and mutant IBA57 again suggests a detrimental effect of the R147W amino acid exchange on mitochondrial [4Fe-4S] cluster assembly.

A yet ill-explained hallmark of IBA57 deficiency is the decrease in RCC-IV (COX) activity (Sheftel et al., 2012; Ajit Bolar et al., 2013; Debray et al., 2015; Stehling et al., 2018b), which is associated with a decline of COX2 and COX6A/B subunit levels (Figures 3C, E, F). Complementation by wild-type smIBA57 restored both RCC-IV activity and subunit levels, but smIBA57^R147W^ only recovered the COX enzymatic activity to some extent, while the COX2 and COX6A/B levels remained diminished (Sheftel et al., 2012). By contrast, neither RNAi-mediated depletion of IBA57 nor complementation by smIBA57^wt^ or smIBA57^R147W^ substantially affected the [2Fe-2S] subunit UQCRFS1 (Rieske Fe/S protein) of RCC-III (Supplementary Figure S2E), which is consistent with the specific function of IBA57 in the maturation of mitochondrial [4Fe-4S] but not of [2Fe/2S] proteins (Sheftel et al., 2012). Accordingly, the UQCRC2 subunit level also remained unchanged. Immunostaining against the F1α/β subunits of complex V (F_1_F_o_-ATP synthase) did not reveal any alterations (Figures 3C, E, F) as well, arguing against a general OXPHOS defect caused by the IBA57-R147W amino acid exchange. Overall, these results are fully consistent with an MMDS3-related phenotype in the HNM-affected Kooiker dogs.

Unlike the core ISC-mediated [2Fe-2S] cluster assembly process, the synthesis of mitochondrial [4Fe-4S] proteins do not generally interfere with the cofactor status of extra-mitochondrial Fe/S proteins, except for some indirect, ROS-mediated effects on iron regulatory protein 1 (IRP1) (Navarro-Sastre et al., 2011; Sheftel et al., 2012; Uzarska et al., 2013; Wang et al., 2022). Accordingly, RNAi-mediated depletion of IBA57 exerted subtle effects on the cytosolic aconitase activity of IRP1 in comparison to the non-Fe/S enzyme lactate dehydrogenase (LDH; Supplementary Figure S3A, B), while the levels of IRP1 protein remained unaffected (Supplementary Figure S3C–F). Likewise, probing for the amounts of several cytosolic and nuclear Fe/S and control proteins by immunoblotting did not reveal major alterations, with the notable exception of the CIA component CIAO3, which is known to be sensitive to disturbances in mitochondrial homeostasis (Torraco et al., 2017). Taken together, the IBA57-R147W mutant protein only partially and specifically complemented the mitochondrial [4Fe-4S] protein defects elicited by IBA57 depletion. Collectively, the findings strongly suggest that the *IBA57* Kooiker dog mutation is causative for the observed HNM phenotype by impairing the function of canine IBA57 in mitochondrial [4Fe-4S] protein assembly.

## Discussion

We have shown here, by genetic association and linkage analysis, that the gene causing HNM in Kooiker dogs is located near the centromere of chromosome 14. The *p*-value obtained for the association of this region was almost 4 orders of magnitude lower than the *p*-value for the next best region (Figure 1). The LOD score of 6.1 was well above the threshold value of 3, generally considered the proof of linkage.

Two of the 38 genes in the HNM candidate region are known for causing neurodegenerative phenotypes in humans. First, mutations in human *GJC2*, coding for gap junction protein gamma-2 (OMIM 608803), are the cause of hypomyelinating leukodystrophy 2 (OMIM 608804), lymphatic malformation 3 (OMIM 613480), and autosomal recessive spastic paraplegia 44 (OMIM 613206). However, no mutations were found in the coding DNA sequences of *GJC2* of affected Kooiker dogs. Second, mutations in human *IBA57* have been implicated in neurological diseases such as spastic paraplegia 74 (OMIM 615316) and multiple mitochondrial dysfunctions syndrome 3 (MMDS3, OMIM 615330) (Ajit Bolar et al., 2013; Debray et al., 2015; Lossos et al., 2015; Ishiyama et al., 2017; Lebigot et al., 2017; Torraco et al., 2017; Hamanaka et al., 2018; Liu et al., 2018). We identified a XM_025454932:c.439C>T mutation in canine *IBA57* that co-segregated with HNM in the Kooiker dog pedigrees. The resulting XP_025310717:R147W mutation affected the second of two consecutive arginine residues that are conserved in animals but not in other species (Figure 2).

IBA57 is essential for the assembly of [4Fe-4S] clusters in the mitochondria and cooperates with the mitochondrial ISC factors ISCA1 and ISCA2. Consistent with the tight functional cooperation of these three proteins, mutations in their genes have been associated with similar disease phenotypes summarized as MMDS (Ajit Bolar et al., 2013; Debray et al., 2015; Lossos et al., 2015; Ishiyama et al., 2017; Lebigot et al., 2017; Torraco et al., 2017; Hamanaka et al., 2018; Liu et al., 2018). Mutations in human *IBA57* seem to occur more frequently than in the other two genes, and the phenotypical spectrum spans from relatively mild neurological symptoms to fatal outcome at birth. The severity of the disease cases correlates well with the associated specific biochemical defects in mitochondrial [4Fe-4S] proteins. Due to the neurodegenerative consequences of dysfunctional IBA57, the IBA57 c.439C>T mutation appeared to us as the likely candidate for the cause of HNM, yet direct biochemical testing in dog material was not feasible.

In order to characterize the Kooiker dog IBA57 amino acid exchange R147W in more detail, we applied an established depletion–complementation assay in human tissue culture (Ajit Bolar et al., 2013; Debray et al., 2015; Lossos et al., 2015). Endogenous IBA57 was depleted in HeLa cells by RNAi, followed by plasmid-based expression of the mutant protein IBA57-R147W carrying the canine mutation at the homologous position. Transfection with the plasmid containing mutant *IBA57* resulted in markedly less efficient complementation of the IBA57 deficiency phenotypes than the expression of wild-type *IBA57*, unequivocally demonstrating that the HNM-related R147W amino acid exchange strongly compromised the activity of IBA57 in the mitochondrial [4Fe-4S] cluster assembly. The strongest defects were observed in the integrity and activity of respiratory chain complexes I and II (SDH), as well as in LIAS-dependent lipoyl cofactor formation on the pyruvate dehydrogenase complex subunit PDHc-E2. In addition, an MMDS3-typical deterioration of respiratory chain complex IV (Sheftel et al., 2012; Ajit Bolar et al., 2013; Debray et al., 2015; Stehling et al., 2018b) was observed, collectively resulting in a severe impairment of mitochondrial energy metabolism. The complex IV (COX) defects generally observed upon depletion of the ISC components, which includes the ISCA and IBA57 proteins, still lack a molecular explanation (Lill and Freibert, 2020). Either this impairment is indirectly caused by defects in the supercomplex partners RCC-I and RCC-II. Alternatively, it may be explained by a maturation defect of an uncharacterized mitochondrial [4Fe-4S] protein involved in, e.g., subunit synthesis or assembly of COX. In line with the specific function of IBA57 in mitochondrial [4Fe-4S] but not [2Fe-2S] cluster formation (Sheftel et al., 2012; Lill and Freibert, 2020), the [2Fe-2S] cofactor–containing RCC-III, as well as extra-mitochondrial Fe/S proteins, is not significantly affected by the HNM-related mutation.

Whereas the IBA57-R147W substitution has not been described before as pathogenic, two substitutions of the preceding conserved arginine residue either to tryptophan (R146W) or to proline (R146P) were found homozygous or compound heterozygous, respectively, in human patients with MMDS3 (Zeng et al., 2014; Weiler et al., 2020). Plasmid-based expression of human IBA57-R147W did not indicate that the amino acid substitution substantially affected the protein’s stability, similar to the human disease-causing Trp substitution of the neighboring Arg146 (Debray et al., 2015). Both arginine residues are a part of the surface-exposed loop located not far from IBA57’s conserved active site cysteine (Debray et al., 2015; Camponeschi et al., 2022). Structural modeling of the R146W substitution has proposed that the alteration might interfere with the interaction of IBA57 and its partner protein ISCA2 (Camponeschi et al., 2022), and this might decrease the efficiency of the [4Fe-4S] cluster assembly. In contrast to the neighboring Arg146, residue Arg147 appears to hardly protrude from IBA57 (Gourdoupis et al., 2018) and might thus be less relevant for interactions with ISCA2, explaining why in contrast to the fatal human R146W amino acid exchange, the present HNM-related R147W substitution elicits a rather mild phenotype. The fact that this residue is not conserved in, e.g., plants, fungi, and bacteria further fits the relatively mild consequences caused by this mutation. Although other human IBA57 alterations such as Thr106Ala (Lebigot et al., 2017; Torraco et al., 2017), Tyr108Ser (Ishiyama et al., 2017), Leu112Ser (Lebigot et al., 2017), Trp196Gly, Pro229Leu (Torraco et al., 2017), and Gln314Pro (Ajit Bolar et al., 2013) are also less surface exposed or even buried (Camponeschi et al., 2022), they elicit major functional IBA57 deficiencies such as low protein levels, resulting in severe disease phenotypes.

In addition to the *IBA57* mutation, we found a nonsense mutation in *OBSCN*, which also co-segregated with HNM to some extent. Large obscurin isoforms are only found as constituents of the sarcomeric cytoskeleton of striated muscle cells. Smaller obscurins have been detected in several rat and mouse tissues, but the significance of these is not clear (Ackermann et al., 2018). Frameshift mutations in the central part of human *OBSCN* have been implicated in cardiomyopathies with an apparent dominant effect (Grogan and Kontrogianni-Konstantopoulos, 2019). Over the years, about 10% of the Kooiker dog population have been heterozygous carriers of the *OBSCN* stop codon mutation, yet—according to the meticulously maintained health records of the breed club (Snels, 2022)—this group was not predisposed to cardiomyopathies. Interestingly, several losses of function mutations in human *OBSCN* have recently been shown to be associated with a recessive form of recurrent rhabdomyolysis, clearly distinct from the neurologic phenotype of HNM (Cabrera-Serrano et al., 2022). Therefore, we did not consider the *OBSCN* mutation to be primarily responsible for the HNM phenotype. It is very well possible that dogs that are homozygous for the *OBSCN* mutation would develop rhabdomyolysis at a later age if they would have no *IBA57* mutation. On the other hand, the clinical phenotype of HNM in Kooiker dogs exhibits similarities to the leukoencephalomyelopathy that occurs in Rottweilers (Gamble and Chrisman, 1984; Wouda and van Nes, 1986) and Leonbergers (Oevermann et al., 2008) where the causative mutation has been recently identified in the gene *NAPEPLD* (Minor et al., 2018). However, *NAPEPLD* is involved in the synthesis of N-acylethanolamines and not in the Fe/S protein assembly, excluding any direct involvement in the observed biochemical alterations.

Human patients with an IBA57 deficiency suffering from a leukodystrophy-like phenotype were first described in 2013 (Ajit Bolar et al., 2013). Since then, a variety of deleterious *IBA57* mutations have been identified in multiple individuals (Camponeschi et al., 2022), now commonly subsumed to MMDS3. The severity of MMDS3 varies widely and is probably related to the level of residual IBA57 activity afforded by gene alterations, although large phenotypic variability has also been observed in two siblings with the same *IBA57* mutations (Hamanaka et al., 2018). Based on the clinical phenotype, human MMDS3 patients may be classified into three categories (Liu et al., 2018): first is the most severe congenital type, leading to perinatal death and only observed in the two siblings; second is the most prevalent infantile type, with an age at onset of at least 4 months and variable outcome; and third is the comparably least affected childhood-type, with an age at onset of at least 3 years, slowly progressing spastic paraplegia, optic atrophy, and peripheral polyneuropathy, summarized as the SPOAN-like phenotype (Lossos et al., 2015). The childhood type was only observed in a consanguineous, multiplex family and linked to a homozygous splice site mutation leading to a low-level expression of the non-mutated IBA57 protein. The clinical manifestation of the affected Kooiker dogs appears to be as mild as in the human childhood type, suggesting that the R147W amino acid exchange affected the function of IBA57 only moderately. However, the genetic alterations causing the human SPOAN-like and the canine HNM phenotype are not comparable, which may explain why optic atrophy is not observed in dogs. The MRI examinations of human patients invariably show lesions in the white matter of the brain and sometimes of the spinal cord. In the Kooiker dogs, the white matter lesions are mainly seen in the spinal cord and rarely in the brain. This divergence may reflect different energy requirements or a dedicated vulnerability of different parts of the central nervous system, particularly among man and dog. Overall, species specificity may also explain some of these subtle differences.

## Data Availability

The data sets presented in this study can be found in online repositories. The names of the repository/repositories and accession number(s) can be found at https://www.ncbi.nlm.nih.gov/, PRJNA948354.

## Footnotes

1 Health theme of the Seventh Framework Program (FP7-LUPA, GA: 201370).
