# Supplementary material for: A novel IBA57 variant is associated with mitochondrial iron–sulfur protein deficiency and necrotizing myelopathy in dogs
Source: Front Genet. 2023 Jul 12;14:1190222. doi: 10.3389/fgene.2023.1190222 (PMC10425596; doi:10.3389/fgene.2023.1190222)
Supplement: Supplementary file 1 [file DataSheet1.docx]

**Supplementary Material**

**for**

**A novel IBA57 variant is associated with mitochondrial iron-sulfur protein deficiency and necrotizing myelopathy in dogs**

Paul J.J. Mandigers, Oliver Stehling, Manon Vos-Loohuis, Frank G. van Steenbeek,

Roland Lill, Peter A. Leegwater

**Content:**

Supplementary Figures 1-3

Supplementary Tables 1-3

Supplementary References


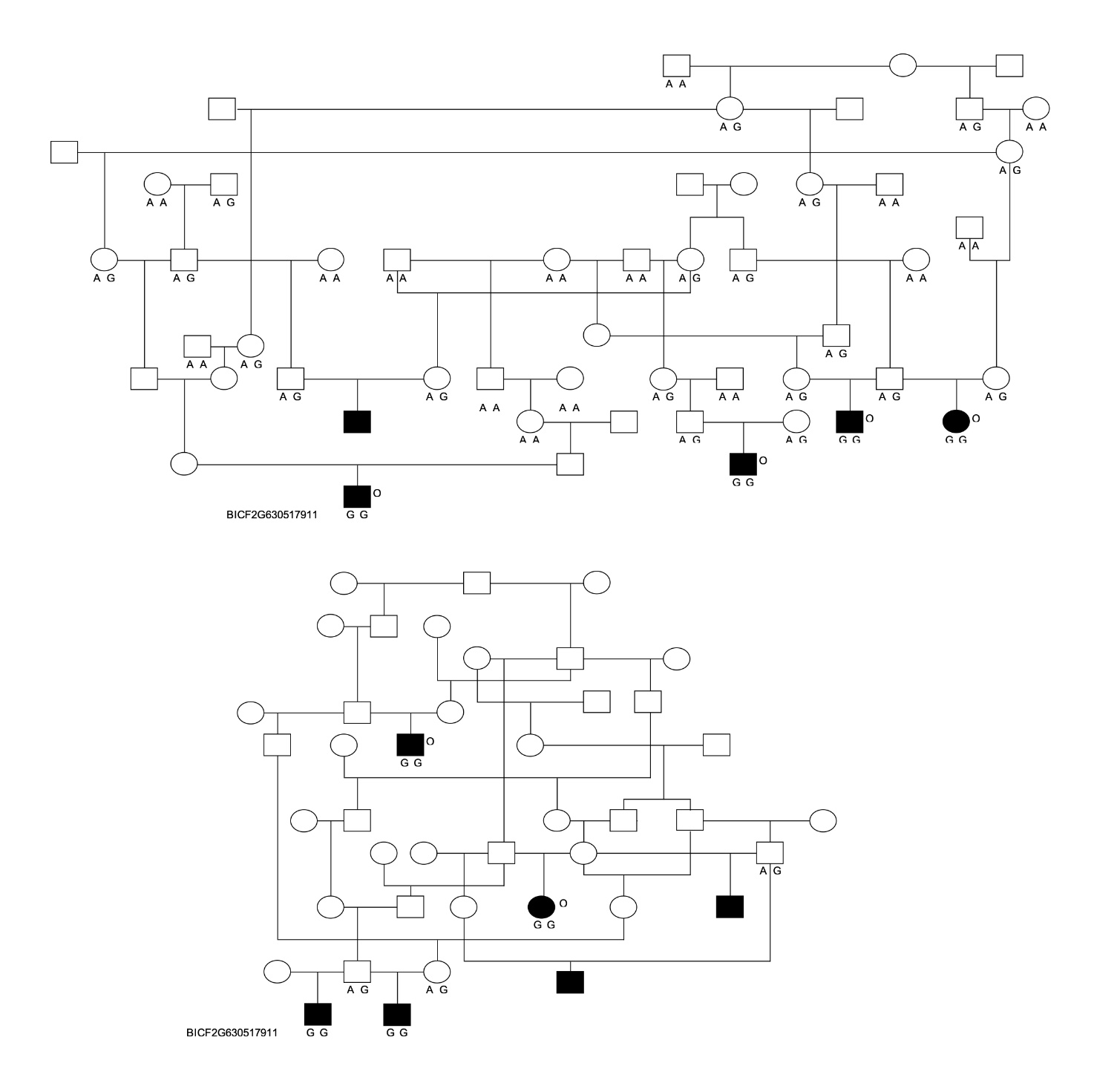


**Supplementary Figure S1. Co-segregation of Hereditary Necrotizing Myelopathy with SNP BICF2G630517911.**

The available samples of 2 pedigrees were genotyped for the marker at Chr14:3021087 (CanFam 3.1) by dideoxy DNA sequencing. The LOD score for linkage between the G allele of the SNP and HNM was 6.1 as calculated with Superlink. o: included in genome-wide association analysis.


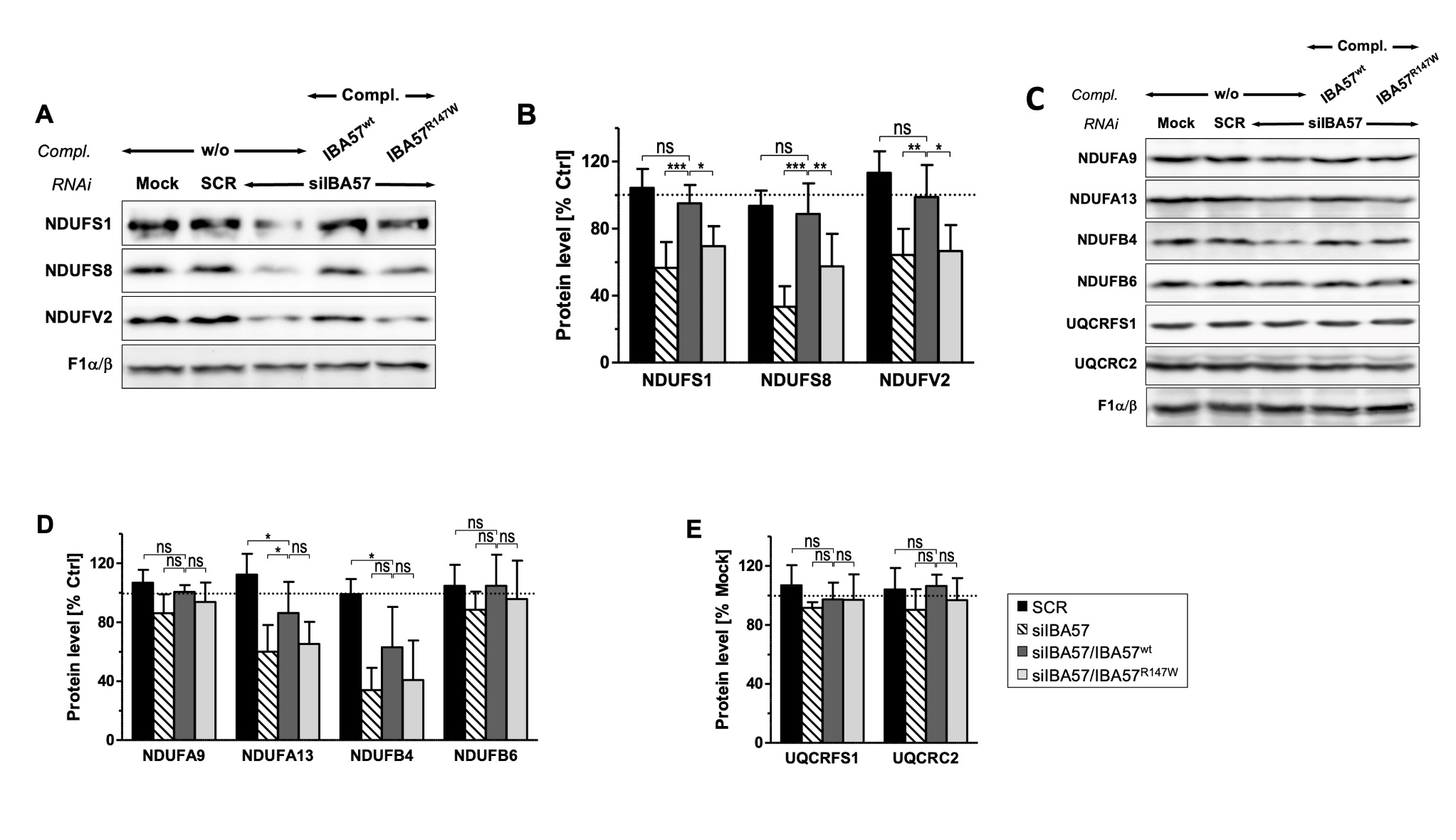
**Supplementary Figure S2. The IBA57-R147W amino acid exchange compromises the stability of RCC-I but not of RCC-III subunits.**

HeLa cells were depleted for IBA57 and treated as in Fig. 3. (A) Total cell lysates were subjected to immunoblotting and analyzed for the steady-state protein levels of the indicated RCC-I Fe/S subunits as well as for F1α/β. (B) Immunoblot signals of the indicated RCC-I subunits from (A) were quantiﬁed relative to β-actin levels (c.f. Fig. 3A), and the ratio was normalized to mock-transfected control cells (dashed line). (C) Steady-state protein levels of indicated RCC-I and RCC-III subunits as well as of F1α/β were analyzed in total cell lysates by immunoblotting. (D,E) Immunoblot signals of RCC-I (D) and RCC-III (E) subunits from (C) were quantiﬁed relative to β-actin levels, and the ratio was normalized to mock-transfected control cells (dashed lines). Representative blots are shown. All values are given as the mean ±SD (n = 3 to 4); * P <0.05; **

P <0.01; *** P <0.001; ns, not signiﬁcant.

**
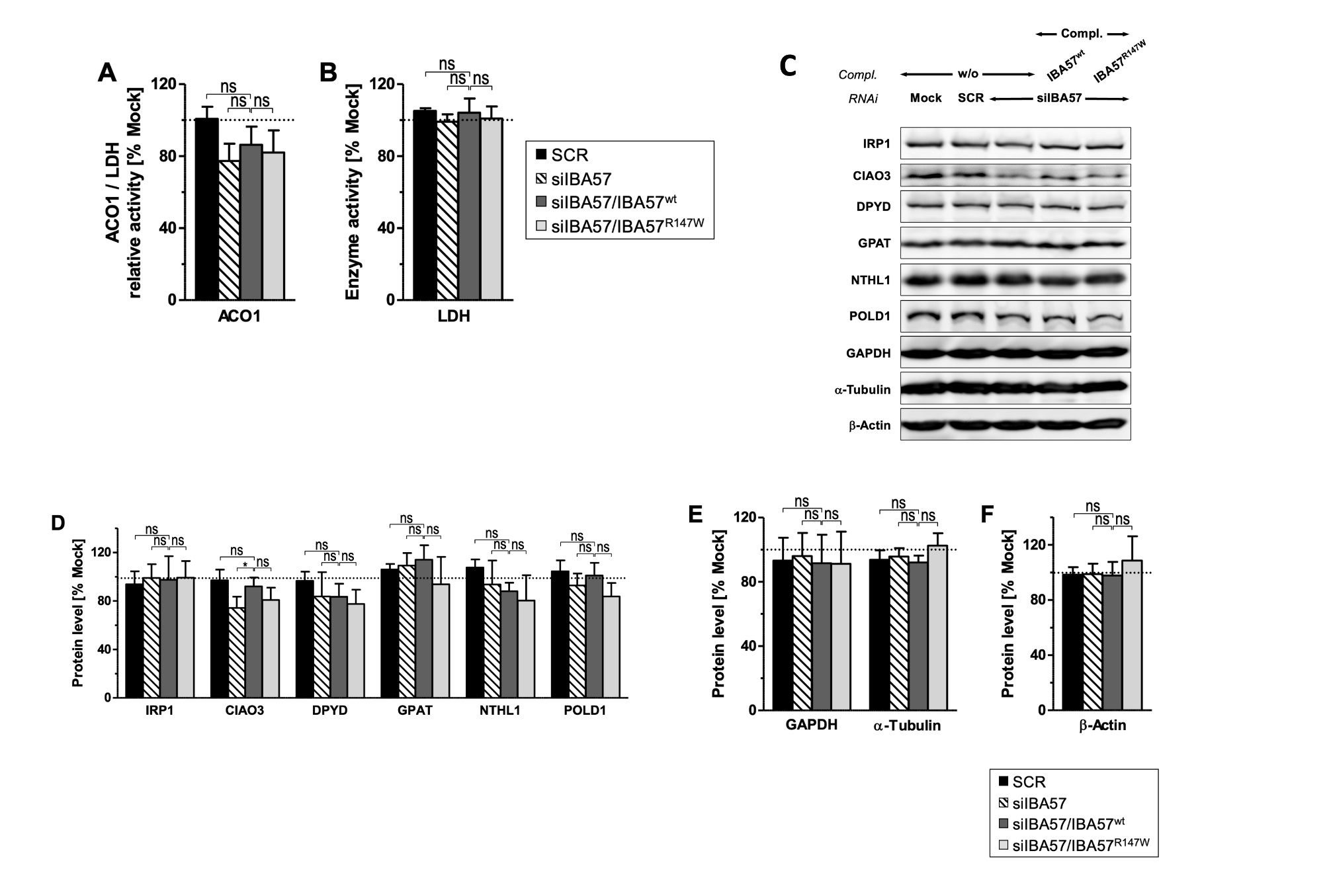
Supplementary Figure S3. The IBA57-R147W amino acid exchange does not generally affect cytosolic or nuclear Fe/S proteins.**

HeLa cells were depleted for IBA57 and treated as in Fig. 3. (A,B) The speciﬁc activity of cytosolic aconitase (ACO1) (A) was determined in cytosolic fractions obtained from digitonin-based plasma membrane permeabilization, related to lactate dehydrogenase (LDH) activity (B), and normalized to mock-transfected control cells (dashed lines). (C) Total cell lysates were subjected to immunoblotting and analyzed for the levels of indicated cytosolic or nuclear proteins. (D-F) Immunoblot signals from part (C) for cytosolic and nuclear Fe/S proteins (D) and reference proteins (E) were quantiﬁed relative to β-actin levels (F) and the ratio was normalized to mock-transfected control cells (dashed lines). Representative blots are shown. All values are given as the mean ±SD (n = 3 to 4); * P <0.05; ** P <0.01; *** P <0.001; ns, not signiﬁcant.

| **Supplementary Table S1. DNA sequences of oligonucleotides for PCR and chain termination sequencing of SNP and gene variants.** | | |
| --- | --- | --- |
|  |  |  |
| oligonucleotide | variant position | sequence |
| BICF2G630517911_FW | 14:3021087 | ATATTCTTGATCTGCCCATGCC |
| BICF2G630517911_RV | 14:3021087 | TTTCAGCTTCAAGTGTTGTGG |
| IBA57_FW | 14:801179 | CTCAGTGGACGTGAGGTC |
| IBA57_RV | 14:801179 | GAGCTAGCACTGAACACCT |
| OBSCN_FW | 14:693260 | AGCGATGAGGAATCCAAGAC |
| OBSCN_RV | 14:693260 | GCCATCCTGAGATTCCATAGAG |

| **Supplementary Table S2. Curation of DNA sequence variants as candidates for hereditary necrotizing myelopathy in Kooiker dogs.** | | | | | | | |  |  |  |
| --- | --- | --- | --- | --- | --- | --- | --- | --- | --- | --- |
| #CHROM | POS | ID | REF | ALT | QUAL | FILTER | INFO |  | Curation | |
| 14 | 467539 | . | C | A | 2241.32 | PASS | AC=5;AF=0.357;AN=14;BaseQRankSum=1.038;ClippingRankSum=0.595;DP=213;FS=5.496;MLEAC=5;MLEAF=0.357;MQ=60.00;MQRankSum=0.348;QD=23.59;ReadPosRankSum=2.323;SOR=1.090;ANN=A\|missense_variant\|MODERATE\|ENSCAFG00000024874\|ENSCAFG00000024874\|transcript\|ENSCAFT00000001758\|protein_coding\|11/11\|c.1324G>T\|p.Asp442Tyr\|1324/1500\|1324/1500\|442/499\|\|WARNING_TRANSCRIPT_NO_START_CODON | . | 31 dogs of DBVDC homozygous for ALT-allele | |
| 14 | 468009 | . | C | T | 1865.29 | PASS | AC=4;AF=0.286;AN=14;BaseQRankSum=2.280;ClippingRankSum=-2.706;DP=157;FS=10.918;MLEAC=4;MLEAF=0.286;MQ=60.00;MQRankSum=1.232;QD=26.65;ReadPosRankSum=1.107;SOR=0.220;ANN=T\|missense_variant\|MODERATE\|ENSCAFG00000024874\|ENSCAFG00000024874\|transcript\|ENSCAFT00000001758\|protein_coding\|8/11\|c.920G>A\|p.Gly307Glu\|920/1500\|920/1500\|307/499\|\|WARNING_TRANSCRIPT_NO_START_CODON | . | 33 dogs of DBVDC homozygous for ALT-allele | |
| 14 | 507272 | . | GA | G | 723.34 | PASS | AC=4;AF=0.333;AN=12;BaseQRankSum=-0.081;ClippingRankSum=1.413;DP=105;FS=9.512;MLEAC=4;MLEAF=0.333;MQ=59.92;MQRankSum=-0.454;QD=19.04;ReadPosRankSum=1.939;SOR=1.280;ANN=G\|frameshift_variant\|HIGH\|ENSCAFG00000001138\|ENSCAFG00000001138\|transcript\|ENSCAFT00000001763\|protein_coding\|5/8\|c.786delA\|p.Lys262fs\|786/1512\|786/1512\|262/503\|\|WARNING_TRANSCRIPT_NO_START_CODON&INFO_REALIGN_3_PRIME;LOF=(ENSCAFG00000001138\|ENSCAFG00000001138\|1\|1.00) | . | microsatellite repeat in wrongly predicted exon | |
| 14 | 507336 | . | G | GA,GAAAGAAAGA | 469.02 | PASS | AC=2,3;AF=0.167,0.250;AN=12;BaseQRankSum=-0.849;ClippingRankSum=0.580;DP=90;FS=0.000;MLEAC=2,2;MLEAF=0.167,0.167;MQ=57.23;MQRankSum=-3.561;QD=7.33;ReadPosRankSum=-0.849;SOR=0.862;ANN=GA\|frameshift_variant\|HIGH\|ENSCAFG00000001138\|ENSCAFG00000001138\|transcript\|ENSCAFT00000001763\|protein_coding\|5/8\|c.843dupA\|p.Glu282fs\|844/1512\|844/1512\|282/503\|\|WARNING_TRANSCRIPT_NO_START_CODON&INFO_REALIGN_3_PRIME,GAAAGAAAGA\|inframe_insertion\|MODERATE\|ENSCAFG00000001138\|ENSCAFG00000001138\|transcript\|ENSCAFT00000001763\|protein_coding\|5/8\|c.843_844insAGAAAGAAA\|p.Glu281_Glu282insArgLysLys\|844/1512\|844/1512\|282/503\|\|WARNING_TRANSCRIPT_NO_START_CODON&INFO_REALIGN_3_PRIME;LOF=(ENSCAFG00000001138\|ENSCAFG00000001138\|1\|1.00) | . | microsatellite repeat in wrongly predicted exon | |
| 14 | 507339 | . | G | GA | 162.79 | PASS | AC=2;AF=0.167;AN=12;BaseQRankSum=-1.540;ClippingRankSum=0.775;DP=93;FS=0.000;MLEAC=2;MLEAF=0.167;MQ=56.94;MQRankSum=-3.385;QD=16.28;ReadPosRankSum=-0.971;SOR=1.022;ANN=GA\|frameshift_variant\|HIGH\|ENSCAFG00000001138\|ENSCAFG00000001138\|transcript\|ENSCAFT00000001763\|protein_coding\|5/8\|c.846dupA\|p.Glu283fs\|847/1512\|847/1512\|283/503\|\|WARNING_TRANSCRIPT_NO_START_CODON&INFO_REALIGN_3_PRIME;LOF=(ENSCAFG00000001138\|ENSCAFG00000001138\|1\|1.00) | . | microsatellite repeat in wrongly predicted exon | |
| 14 | 507357 | . | G | GA | 302.68 | PASS | AC=4;AF=0.286;AN=14;BaseQRankSum=-0.188;ClippingRankSum=1.048;DP=98;FS=3.189;MLEAC=4;MLEAF=0.286;MQ=56.90;MQRankSum=-3.926;QD=9.46;ReadPosRankSum=1.611;SOR=1.363;ANN=GA\|frameshift_variant\|HIGH\|ENSCAFG00000001138\|ENSCAFG00000001138\|transcript\|ENSCAFT00000001763\|protein_coding\|5/8\|c.864dupA\|p.Glu289fs\|865/1512\|865/1512\|289/503\|\|WARNING_TRANSCRIPT_NO_START_CODON&INFO_REALIGN_3_PRIME;LOF=(ENSCAFG00000001138\|ENSCAFG00000001138\|1\|1.00) | . | microsatellite repeat in wrongly predicted exon | |
| 14 | 507360 | . | G | GA | 708.96 | PASS | AC=6;AF=0.500;AN=12;BaseQRankSum=-0.332;ClippingRankSum=-0.332;DP=101;FS=9.483;MLEAC=6;MLEAF=0.500;MQ=57.00;MQRankSum=-1.115;QD=8.24;ReadPosRankSum=4.481;SOR=1.497;ANN=GA\|frameshift_variant\|HIGH\|ENSCAFG00000001138\|ENSCAFG00000001138\|transcript\|ENSCAFT00000001763\|protein_coding\|5/8\|c.867dupA\|p.Glu290fs\|868/1512\|868/1512\|290/503\|\|WARNING_TRANSCRIPT_NO_START_CODON&INFO_REALIGN_3_PRIME;LOF=(ENSCAFG00000001138\|ENSCAFG00000001138\|1\|1.00) | . | microsatellite repeat in wrongly predicted exon | |
| 14 | 510232 | . | A | C | 1686.32 | PASS | AC=5;AF=0.357;AN=14;BaseQRankSum=-0.891;ClippingRankSum=-0.082;DP=154;FS=9.180;MLEAC=5;MLEAF=0.357;MQ=60.00;MQRankSum=0.150;QD=26.35;ReadPosRankSum=0.831;SOR=0.246;ANN=C\|synonymous_variant\|LOW\|ENSCAFG00000001138\|ENSCAFG00000001138\|transcript\|ENSCAFT00000001763\|protein_coding\|8/8\|c.1506A>C\|p.Gly502Gly\|1506/1512\|1506/1512\|502/503\|\|WARNING_TRANSCRIPT_NO_START_CODON | . | synonymous variant | |
| 14 | 599485 | . | C | T | 443.7 | PASS | AC=2;AF=0.143;AN=14;BaseQRankSum=-0.440;ClippingRankSum=0.465;DP=125;FS=2.555;MLEAC=2;MLEAF=0.143;MQ=51.18;MQRankSum=0.824;QD=34.13;ReadPosRankSum=1.509;SOR=0.616;ANN=T\|synonymous_variant\|LOW\|ENSCAFG00000024336\|ENSCAFG00000024336\|transcript\|ENSCAFT00000037582\|protein_coding\|1/1\|c.78C>T\|p.Asp26Asp\|78/381\|78/381\|26/126\|\|WARNING_TRANSCRIPT_NO_START_CODON,T\|downstream_gene_variant\|MODIFIER\|ENSCAFG00000024333\|ENSCAFG00000024333\|transcript\|ENSCAFT00000037577\|protein_coding\|\|c.*381G>A\|\|\|\|\|4331\| | . | synonymous variant | |
| 14 | 646656 | . | T | C | 1083.71 | PASS | AC=3;AF=0.214;AN=14;BaseQRankSum=1.228;ClippingRankSum=0.696;DP=175;FS=2.741;MLEAC=3;MLEAF=0.214;MQ=60.00;MQRankSum=0.517;QD=27.58;ReadPosRankSum=0.517;SOR=0.585;ANN=C\|synonymous_variant\|LOW\|TRIM11\|ENSCAFG00000001145\|transcript\|ENSCAFT00000001778\|protein_coding\|6/6\|c.1389T>C\|p.Asp463Asp\|1389/4111\|1389/1407\|463/468\|\| | . | synonymous variant | |
| 14 | 647754 | . | C | T | 1087.72 | PASS | AC=3;AF=0.214;AN=14;BaseQRankSum=0.319;ClippingRankSum=-1.676;DP=133;FS=2.748;MLEAC=3;MLEAF=0.214;MQ=60.00;MQRankSum=-2.869;QD=26.11;ReadPosRankSum=1.360;SOR=1.000;ANN=T\|3_prime_UTR_variant\|MODIFIER\|TRIM11\|ENSCAFG00000001145\|transcript\|ENSCAFT00000001778\|protein_coding\|6/6\|c.*1080C>T\|\|\|\|\|1080\| | . | 3-prime UTR | |
| 14 | 647850 | . | C | T | 718.71 | PASS | AC=3;AF=0.214;AN=14;BaseQRankSum=0.920;ClippingRankSum=1.060;DP=124;FS=2.091;MLEAC=3;MLEAF=0.214;MQ=60.00;MQRankSum=-0.584;QD=31.25;ReadPosRankSum=-0.234;SOR=0.612; ANN=T\|3_prime_UTR_variant\|MODIFIER\|TRIM11\|ENSCAFG00000001145\|transcript\|ENSCAFT00000001778\|protein_coding\|6/6\|c.*1176C>T\|\|\|\|\|1176\| | . | 3-prime UTR | |
| 14 | 648488 | . | TC | T | 198.51 | PASS | AC=5;AF=0.500;AN=10;BaseQRankSum=0.038;ClippingRankSum=-1.398;DP=31;FS=14.880;MLEAC=5;MLEAF=0.500;MQ=60.00;MQRankSum=0.416;QD=7.63;ReadPosRankSum=1.323;SOR=4.804;ANN=T\|3_prime_UTR_variant\|MODIFIER\|TRIM11\|ENSCAFG00000001145\|transcript\|ENSCAFT00000001778\|protein_coding\|6/6\|c.*1824delC\|\|\|\|\|1824\|INFO_REALIGN_3_PRIME | . | 3-prime UTR | |
| 14 | 668951 | . | T | C | 1170.09 | PASS | AC=4;AF=0.286;AN=14;BaseQRankSum=-0.235;ClippingRankSum=-0.395;DP=143;FS=2.594;MLEAC=4;MLEAF=0.286;MQ=60.00;MQRankSum=-0.627;QD=21.67;ReadPosRankSum=1.073;SOR=0.953;ANN=C\|synonymous_variant\|LOW\|OBSCN\|ENSCAFG00000001161\|transcript\|ENSCAFT00000050108\|protein_coding\|106/117\|c.22002A>G\|p.Thr7334Thr\|22033/23803\|22002/23772\|7334/7923\|\| | . | synonymous variant | |
| 14 | 690052 | . | C | T | 750.49 | PASS | AC=4;AF=0.286;AN=14;BaseQRankSum=2.721;ClippingRankSum=-1.703;DP=67;FS=2.457;MLEAC=4;MLEAF=0.286;MQ=60.00;MQRankSum=-0.417;QD=21.44;ReadPosRankSum=1.618;SOR=0.446;ANN=T\|synonymous_variant\|LOW\|OBSCN\|ENSCAFG00000001161\|transcript\|ENSCAFT00000050108\|protein_coding\|85/117\|c.17940G>A\|p.Ala5980Ala\|17971/23803\|17940/23772\|5980/7923\|\|,T\|synonymous_variant\|LOW\|OBSCN\|ENSCAFG00000001161\|transcript\|ENSCAFT00000001792\|protein_coding\|67/74\|c.16350G>A\|p.Ala5450Ala\|16381/17248\|16350/17217\|5450/5738\|\| | . | synonymous variant | |
| 14 | 690418 | . | A | G | 1653.15 | PASS | AC=6;AF=0.429;AN=14;BaseQRankSum=-0.973;ClippingRankSum=0.226;DP=92;FS=7.693;MLEAC=6;MLEAF=0.429;MQ=60.00;MQRankSum=-0.702;QD=22.65;ReadPosRankSum=-0.062;SOR=0.193;ANN=G\|synonymous_variant\|LOW\|OBSCN\|ENSCAFG00000001161\|transcript\|ENSCAFT00000050108\|protein_coding\|84/117\|c.17763T>C\|p.His5921His\|17794/23803\|17763/23772\|5921/7923\|\|,G\|synonymous_variant\|LOW\|OBSCN\|ENSCAFG00000001161\|transcript\|ENSCAFT00000001792\|protein_coding\|66/74\|c.16173T>C\|p.His5391His\|16204/17248\|16173/17217\|5391/5738\|\| | . | synonymous variant | |
| 14 | 690919 | . | A | G | 1493.01 | PASS | AC=7;AF=0.500;AN=14;BaseQRankSum=-0.513;ClippingRankSum=1.120;DP=89;FS=4.034;MLEAC=7;MLEAF=0.500;MQ=60.00;MQRankSum=0.365;QD=20.45;ReadPosRankSum=-0.152;SOR=0.377;ANN=G\|synonymous_variant\|LOW\|OBSCN\|ENSCAFG00000001161\|transcript\|ENSCAFT00000050108\|protein_coding\|82/117\|c.17424T>C\|p.Ala5808Ala\|17455/23803\|17424/23772\|5808/7923\|\|,G\|synonymous_variant\|LOW\|OBSCN\|ENSCAFG00000001161\|transcript\|ENSCAFT00000001792\|protein_coding\|64/74\|c.15834T>C\|p.Ala5278Ala\|15865/17248\|15834/17217\|5278/5738\|\| | . | synonymous variant | |
| 14 | 693260 | . | G | A | 794.72 | PASS | AC=2;AF=0.143;AN=14;BaseQRankSum=-0.655;ClippingRankSum=-0.551;DP=113;FS=0.904;MLEAC=2;MLEAF=0.143;MQ=59.89;MQRankSum=-1.069;QD=29.18;ReadPosRankSum=0.788;SOR=0.821;ANN=A\|stop_gained\|HIGH\|OBSCN\|ENSCAFG00000001161\|transcript\|ENSCAFT00000050108\|protein_coding\|77/117\|c.16774C>T\|p.Arg5592*\|16805/23803\|16774/23772\|5592/7923\|\|,A\|stop_gained\|HIGH\|OBSCN\|ENSCAFG00000001161\|transcript\|ENSCAFT00000001792\|protein_coding\|59/74\|c.15184C>T\|p.Arg5062*\|15215/17248\|15184/17217\|5062/5738\|\|;LOF=(OBSCN\|ENSCAFG00000001161\|2\|1.00);NMD=(OBSCN\|ENSCAFG00000001161\|2\|1.00) | . | OBSCN stop gained | |
| 14 | 693579 | . | A | C | 2076.31 | PASS | AC=6;AF=0.429;AN=14;BaseQRankSum=0.903;ClippingRankSum=0.551;DP=111;FS=0.718;MLEAC=6;MLEAF=0.429;MQ=60.00;MQRankSum=-0.089;QD=22.57;ReadPosRankSum=1.697;SOR=0.751;ANN=C\|missense_variant\|MODERATE\|OBSCN\|ENSCAFG00000001161\|transcript\|ENSCAFT00000050108\|protein_coding\|76/117\|c.16564T>G\|p.Ser5522Ala\|16595/23803\|16564/23772\|5522/7923\|\|,C\|missense_variant\|MODERATE\|OBSCN\|ENSCAFG00000001161\|transcript\|ENSCAFT00000001792\|protein_coding\|58/74\|c.14974T>G\|p.Ser4992Ala\|15005/17248\|14974/17217\|4992/5738\|\| | . | 176 dogs of DBVDC homozygous for ALT-allele | |
| 14 | 694114 | . | A | G | 726.46 | PASS | AC=3;AF=0.214;AN=14;BaseQRankSum=2.183;ClippingRankSum=0.968;DP=93;FS=3.837;MLEAC=3;MLEAF=0.214;MQ=60.00;MQRankSum=0.783;QD=30.14;ReadPosRankSum=0.579;SOR=0.328;ANN=G\|missense_variant\|MODERATE\|OBSCN\|ENSCAFG00000001161\|transcript\|ENSCAFT00000050108\|protein_coding\|75/117\|c.16505T>C\|p.Leu5502Pro\|16536/23803\|16505/23772\|5502/7923\|\|,G\|missense_variant\|MODERATE\|OBSCN\|ENSCAFG00000001161\|transcript\|ENSCAFT00000001792\|protein_coding\|57/74\|c.14918T>C\|p.Leu4973Pro\|14949/17248\|14918/17217\|4973/5738\|\| | . | 1 dog of DBVDC homozygous for ALT-allele | |
| 14 | 696108 | . | C | T | 700.69 | PASS | AC=2;AF=0.143;AN=14;BaseQRankSum=1.300;ClippingRankSum=1.166;DP=139;FS=2.108;MLEAC=2;MLEAF=0.143;MQ=60.00;MQRankSum=-0.382;QD=29.77;ReadPosRankSum=-0.453;SOR=1.044;ANN=T\|missense_variant\|MODERATE\|OBSCN\|ENSCAFG00000001161\|transcript\|ENSCAFT00000050108\|protein_coding\|70/117\|c.15937G>A\|p.Val5313Met\|15968/23803\|15937/23772\|5313/7923\|\|,T\|missense_variant\|MODERATE\|OBSCN\|ENSCAFG00000001161\|transcript\|ENSCAFT00000001792\|protein_coding\|52/74\|c.14350G>A\|p.Val4784Met\|14381/17248\|14350/17217\|4784/5738\|\| | . | 1 dog of DBVDC homozygous for ALT-allele | |
| 14 | 702089 | . | G | A | 627.11 | PASS | AC=2;AF=0.167;AN=12;BaseQRankSum=2.089;ClippingRankSum=0.496;DP=105;FS=1.079;MLEAC=2;MLEAF=0.167;MQ=60.00;MQRankSum=0.897;QD=30.73;ReadPosRankSum=1.871;SOR=0.928;ANN=A\|missense_variant\|MODERATE\|OBSCN\|ENSCAFG00000001161\|transcript\|ENSCAFT00000050108\|protein_coding\|66/117\|c.15323C>T\|p.Ala5108Val\|15354/23803\|15323/23772\|5108/7923\|\|,A\|missense_variant\|MODERATE\|OBSCN\|ENSCAFG00000001161\|transcript\|ENSCAFT00000001792\|protein_coding\|48/74\|c.13736C>T\|p.Ala4579Val\|13767/17248\|13736/17217\|4579/5738\|\| | . | 3 dogs of DBVDC homozygous for ALT-allele | |
| 14 | 702679 | . | T | C | 2406.15 | PASS | AC=6;AF=0.429;AN=14;BaseQRankSum=-4.646;ClippingRankSum=-0.085;DP=147;FS=3.038;MLEAC=6;MLEAF=0.429;MQ=60.00;MQRankSum=1.138;QD=18.65;ReadPosRankSum=-0.790;SOR=0.950;ANN=C\|synonymous_variant\|LOW\|OBSCN\|ENSCAFG00000001161\|transcript\|ENSCAFT00000050108\|protein_coding\|66/117\|c.14733A>G\|p.Ala4911Ala\|14764/23803\|14733/23772\|4911/7923\|\|,C\|synonymous_variant\|LOW\|OBSCN\|ENSCAFG00000001161\|transcript\|ENSCAFT00000001792\|protein_coding\|48/74\|c.13146A>G\|p.Ala4382Ala\|13177/17248\|13146/17217\|4382/5738\|\| | . | synonymous variant | |
| 14 | 712778 | . | G | A | 2077.31 | PASS | AC=6;AF=0.429;AN=14;BaseQRankSum=0.734;ClippingRankSum=-0.538;DP=100;FS=3.929;MLEAC=6;MLEAF=0.429;MQ=60.00;MQRankSum=-1.381;QD=23.61;ReadPosRankSum=0.601;SOR=0.373;ANN=A\|synonymous_variant\|LOW\|OBSCN\|ENSCAFG00000001161\|transcript\|ENSCAFT00000050108\|protein_coding\|59/117\|c.12711C>T\|p.Ile4237Ile\|12742/23803\|12711/23772\|4237/7923\|\|,A\|synonymous_variant\|LOW\|OBSCN\|ENSCAFG00000001161\|transcript\|ENSCAFT00000001792\|protein_coding\|41/74\|c.11031C>T\|p.Ile3677Ile\|11062/17248\|11031/17217\|3677/5738\|\| | . | synonymous variant | |
| 14 | 719284 | . | G | A | 1178.31 | PASS | AC=6;AF=0.429;AN=14;BaseQRankSum=0.141;ClippingRankSum=-1.822;DP=133;FS=0.732;MLEAC=6;MLEAF=0.429;MQ=53.30;MQRankSum=-1.852;QD=13.86;ReadPosRankSum=2.271;SOR=0.582;ANN=A\|synonymous_variant\|LOW\|OBSCN\|ENSCAFG00000001161\|transcript\|ENSCAFT00000050108\|protein_coding\|47/117\|c.10296C>T\|p.Cys3432Cys\|10327/23803\|10296/23772\|3432/7923\|\|,A\|intron_variant\|MODIFIER\|OBSCN\|ENSCAFG00000001161\|transcript\|ENSCAFT00000001792\|protein_coding\|34/73\|c.9406+1259C>T\|\|\|\|\|\| | . | synonymous variant | |
| 14 | 722105 | . | G | A | 551.13 | PASS | AC=4;AF=0.286;AN=14;BaseQRankSum=-1.058;ClippingRankSum=0.510;DP=97;FS=6.717;MLEAC=3;MLEAF=0.214;MQ=60.00;MQRankSum=1.327;QD=28.15;ReadPosRankSum=0.854;SOR=1.644;ANN=A\|synonymous_variant\|LOW\|OBSCN\|ENSCAFG00000001161\|transcript\|ENSCAFT00000050108\|protein_coding\|44/117\|c.9363C>T\|p.Cys3121Cys\|9394/23803\|9363/23772\|3121/7923\|\|,A\|synonymous_variant\|LOW\|OBSCN\|ENSCAFG00000001161\|transcript\|ENSCAFT00000001792\|protein_coding\|32/74\|c.8682C>T\|p.Cys2894Cys\|8713/17248\|8682/17217\|2894/5738\|\| | . | synonymous variant | |
| 14 | 725257 | . | C | T | 718.71 | PASS | AC=3;AF=0.214;AN=14;BaseQRankSum=1.405;ClippingRankSum=0.575;DP=120;FS=6.743;MLEAC=3;MLEAF=0.214;MQ=60.00;MQRankSum=-0.530;QD=25.95;ReadPosRankSum=0.716;SOR=1.065;ANN=T\|missense_variant\|MODERATE\|OBSCN\|ENSCAFG00000001161\|transcript\|ENSCAFT00000050108\|protein_coding\|40/117\|c.8402G>A\|p.Arg2801Gln\|8433/23803\|8402/23772\|2801/7923\|\|,T\|missense_variant\|MODERATE\|OBSCN\|ENSCAFG00000001161\|transcript\|ENSCAFT00000001792\|protein_coding\|28/74\|c.7721G>A\|p.Arg2574Gln\|7752/17248\|7721/17217\|2574/5738\|\| | . | 2 dogs of DBVDC homozygous for ALT-allele | |
| 14 | 729784 | . | C | G | 1748.01 | PASS | AC=7;AF=0.500;AN=14;BaseQRankSum=1.843;ClippingRankSum=0.948;DP=128;FS=4.405;MLEAC=7;MLEAF=0.500;MQ=60.00;MQRankSum=0.610;QD=16.19;ReadPosRankSum=0.759;SOR=1.140;ANN=G\|synonymous_variant\|LOW\|OBSCN\|ENSCAFG00000001161\|transcript\|ENSCAFT00000050108\|protein_coding\|31/117\|c.6279G>C\|p.Gly2093Gly\|6310/23803\|6279/23772\|2093/7923\|\|,G\|synonymous_variant\|LOW\|OBSCN\|ENSCAFG00000001161\|transcript\|ENSCAFT00000001792\|protein_coding\|18/74\|c.5445G>C\|p.Gly1815Gly\|5476/17248\|5445/17217\|1815/5738\|\| | . | synonymous variant | |
| 14 | 731589 | . | G | C | 555.85 | PASS | AC=5;AF=0.417;AN=12;BaseQRankSum=-3.267;ClippingRankSum=-0.197;DP=30;FS=8.671;MLEAC=6;MLEAF=0.500;MQ=60.00;MQRankSum=-0.899;QD=20.59;ReadPosRankSum=-1.338;SOR=0.148;ANN=C\|missense_variant\|MODERATE\|OBSCN\|ENSCAFG00000001161\|transcript\|ENSCAFT00000050108\|protein_coding\|28/117\|c.5602C>G\|p.Leu1868Val\|5633/23803\|5602/23772\|1868/7923\|\|,C\|missense_variant\|MODERATE\|OBSCN\|ENSCAFG00000001161\|transcript\|ENSCAFT00000001792\|protein_coding\|15/74\|c.4768C>G\|p.Leu1590Val\|4799/17248\|4768/17217\|1590/5738\|\| | . | 139 dogs of DBVDC homozygous for ALT-allele | |
| 14 | 732266 | . | C | G | 1484.32 | PASS | AC=8;AF=0.571;AN=14;BaseQRankSum=1.789;ClippingRankSum=1.308;DP=83;FS=3.102;MLEAC=8;MLEAF=0.571;MQ=60.00;MQRankSum=-2.561;QD=20.91;ReadPosRankSum=1.118;SOR=0.384;ANN=G\|missense_variant\|MODERATE\|OBSCN\|ENSCAFG00000001161\|transcript\|ENSCAFT00000050108\|protein_coding\|27/117\|c.5138G>C\|p.Arg1713Pro\|5169/23803\|5138/23772\|1713/7923\|\|,G\|missense_variant\|MODERATE\|OBSCN\|ENSCAFG00000001161\|transcript\|ENSCAFT00000001792\|protein_coding\|14/74\|c.4304G>C\|p.Arg1435Pro\|4335/17248\|4304/17217\|1435/5738\|\| | . | 62 dogs of DBVDC homozygous for ALT-allele | |
| 14 | 751383 | . | T | C | 2041.69 | PASS | AC=6;AF=0.500;AN=12;BaseQRankSum=1.515;ClippingRankSum=1.448;DP=130;FS=2.258;MLEAC=6;MLEAF=0.500;MQ=54.66;MQRankSum=-2.625;QD=18.23;ReadPosRankSum=2.166;SOR=0.495;ANN=C\|synonymous_variant\|LOW\|OBSCN\|ENSCAFG00000001161\|transcript\|ENSCAFT00000050108\|protein_coding\|19/117\|c.3510A>G\|p.Glu1170Glu\|3541/23803\|3510/23772\|1170/7923\|\|,C\|synonymous_variant\|LOW\|OBSCN\|ENSCAFG00000001161\|transcript\|ENSCAFT00000001792\|protein_coding\|9/74\|c.2964A>G\|p.Glu988Glu\|2995/17248\|2964/17217\|988/5738\|\| | . | synonymous variant | |
| 14 | 775660 | . | G | A | 668.11 | PASS | AC=2;AF=0.167;AN=12;BaseQRankSum=1.235;ClippingRankSum=0.044;DP=123;FS=9.491;MLEAC=2;MLEAF=0.167;MQ=60.00;MQRankSum=-1.730;QD=34.51;ReadPosRankSum=-0.044;SOR=0.810;ANN=A\|synonymous_variant\|LOW\|OBSCN\|ENSCAFG00000001161\|transcript\|ENSCAFT00000050108\|protein_coding\|5/117\|c.1902C>T\|p.Leu634Leu\|1933/23803\|1902/23772\|634/7923\|\|,A\|synonymous_variant\|LOW\|OBSCN\|ENSCAFG00000001161\|transcript\|ENSCAFT00000001792\|protein_coding\|5/74\|c.1902C>T\|p.Leu634Leu\|1933/17248\|1902/17217\|634/5738\|\| | . | synonymous variant | |
| 14 | 801179 | . | G | A | 363.16 | PASS | AC=2;AF=0.167;AN=12;BaseQRankSum=-0.768;ClippingRankSum=1.085;DP=93;FS=2.942;MLEAC=2;MLEAF=0.167;MQ=60.00;MQRankSum=0.082;QD=33.01;ReadPosRankSum=0.869;SOR=0.983;ANN=A\|missense_variant\|MODERATE\|IBA57\|ENSCAFG00000001167\|transcript\|ENSCAFT00000001809\|protein_coding\|1/2\|c.100C>T\|p.Arg34Trp\|100/732\|100/732\|34/243\|\|WARNING_TRANSCRIPT_NO_START_CODON | . | IBA57 Arg147Trp | |
| 14 | 822067 | . | T | C | 128.26 | PASS | AC=2;AF=0.143;AN=14;BaseQRankSum=-0.924;ClippingRankSum=-1.864;DP=56;FS=2.144;MLEAC=2;MLEAF=0.143;MQ=60.00;MQRankSum=0.373;QD=32.07;ReadPosRankSum=-0.438;SOR=1.595;ANN=C\|synonymous_variant\|LOW\|GUK1\|ENSCAFG00000001174\|transcript\|ENSCAFT00000001816\|protein_coding\|3/8\|c.192A>G\|p.Pro64Pro\|817/1555\|192/765\|64/254\|\|,C\|upstream_gene_variant\|MODIFIER\|ENSCAFG00000030870\|ENSCAFG00000030870\|transcript\|ENSCAFT00000048509\|protein_coding\|\|c.-1T>C\|\|\|\|\|3673\|WARNING_TRANSCRIPT_NO_START_CODON | . | synonymous variant | |
| 14 | 832562 | . | C | G | 1245.67 | PASS | AC=6;AF=0.429;AN=14;BaseQRankSum=0.333;ClippingRankSum=-0.415;DP=85;FS=4.558;MLEAC=6;MLEAF=0.429;MQ=60.00;MQRankSum=-1.053;QD=31.14;ReadPosRankSum=1.608;SOR=0.341;ANN=G\|missense_variant\|MODERATE\|C1orf35\|ENSCAFG00000001177\|transcript\|ENSCAFT00000001819\|protein_coding\|3/7\|c.222C>G\|p.Asp74Glu\|357/1450\|222/648\|74/215\|\|,G\|downstream_gene_variant\|MODIFIER\|MRPL55\|ENSCAFG00000001176\|transcript\|ENSCAFT00000038029\|protein_coding\|\|c.*375C>G\|\|\|\|\|2493\|,G\|downstream_gene_variant\|MODIFIER\|ARF1\|ENSCAFG00000010433\|transcript\|ENSCAFT00000016560\|protein_coding\|\|c.*543G>C\|\|\|\|\|2965\| | . | 52 dogs of DBVDC homozygous for ALT-allele | |
| 14 | 833587 | . | A | G | 585.72 | PASS | AC=3;AF=0.214;AN=14;BaseQRankSum=1.023;ClippingRankSum=1.858;DP=95;FS=0.000;MLEAC=3;MLEAF=0.214;MQ=59.88;MQRankSum=0.117;QD=32.54;ReadPosRankSum=0.097;SOR=0.605;ANN=G\|3_prime_UTR_variant\|MODIFIER\|C1orf35\|ENSCAFG00000001177\|transcript\|ENSCAFT00000001819\|protein_coding\|7/7\|c.*49A>G\|\|\|\|\|49\|,G\|downstream_gene_variant\|MODIFIER\|MRPL55\|ENSCAFG00000001176\|transcript\|ENSCAFT00000038029\|protein_coding\|\|c.*375A>G\|\|\|\|\|3518\|,G\|downstream_gene_variant\|MODIFIER\|ARF1\|ENSCAFG00000010433\|transcript\|ENSCAFT00000016560\|protein_coding\|\|c.*543T>C\|\|\|\|\|1940\| | . | 3-prime UTR | |
| 14 | 835646 | . | G | A | 465.07 | PASS | AC=4;AF=0.286;AN=14;BaseQRankSum=1.231;ClippingRankSum=0.062;DP=93;FS=12.673;MLEAC=3;MLEAF=0.214;MQ=59.76;MQRankSum=-1.174;QD=28.65;ReadPosRankSum=1.866;SOR=1.456;ANN=A\|synonymous_variant\|LOW\|ARF1\|ENSCAFG00000010433\|transcript\|ENSCAFT00000016560\|protein_coding\|5/5\|c.424C>T\|p.Leu142Leu\|424/543\|424/543\|142/180\|\|,A\|downstream_gene_variant\|MODIFIER\|C1orf35\|ENSCAFG00000001177\|transcript\|ENSCAFT00000001819\|protein_coding\|\|c.*783G>A\|\|\|\|\|1441\| | . | synonymous variant | |
| 14 | 997801 | . | C | T | 1263.71 | PASS | AC=3;AF=0.214;AN=14;BaseQRankSum=-2.416;ClippingRankSum=-0.126;DP=177;FS=8.132;MLEAC=3;MLEAF=0.214;MQ=60.06;MQRankSum=-0.210;QD=30.66;ReadPosRankSum=0.609;SOR=1.448;ANN=T\|3_prime_UTR_variant\|MODIFIER\|PRSS38\|ENSCAFG00000001184\|transcript\|ENSCAFT00000001834\|protein_coding\|5/5\|c.*537G>A\|\|\|\|\|537\| | . | 3-prime UTR | |
| 14 | 1234007 | . | T | C | 690.69 | PASS | AC=2;AF=0.143;AN=14;BaseQRankSum=0.891;ClippingRankSum=-0.476;DP=148;FS=0.000;MLEAC=2;MLEAF=0.143;MQ=60.00;MQRankSum=0.702;QD=35.83;ReadPosRankSum=0.775;SOR=0.785;ANN=C\|missense_variant\|MODERATE\|OR13G1\|ENSCAFG00000001190\|transcript\|ENSCAFT00000038890\|protein_coding\|1/1\|c.299T>C\|p.Phe100Ser\|299/927\|299/927\|100/308\|\|WARNING_TRANSCRIPT_NO_START_CODON | . | 2 dogs of DBVDC homozygous for ALT-allele | |
| 14 | 1297558 | . | A | G | 2593.32 | PASS | AC=5;AF=0.357;AN=14;BaseQRankSum=-0.769;ClippingRankSum=-1.047;DP=189;FS=2.627;MLEAC=5;MLEAF=0.357;MQ=59.93;MQRankSum=0.834;QD=22.95;ReadPosRankSum=0.758;SOR=0.846;ANN=G\|synonymous_variant\|LOW\|OR2AK2\|ENSCAFG00000001192\|transcript\|ENSCAFT00000001844\|protein_coding\|1/1\|c.282A>G\|p.Glu94Glu\|282/903\|282/903\|94/300\|\|WARNING_TRANSCRIPT_NO_START_CODON | . | synonymous variant | |
| 14 | 1298139 | . | T | C | 1155.11 | PASS | AC=2;AF=0.167;AN=12;BaseQRankSum=-3.544;ClippingRankSum=-0.832;DP=166;FS=0.000;MLEAC=2;MLEAF=0.167;MQ=60.00;MQRankSum=0.916;QD=26.86;ReadPosRankSum=1.504;SOR=0.749;ANN=C\|missense_variant\|MODERATE\|OR2AK2\|ENSCAFG00000001192\|transcript\|ENSCAFT00000001844\|protein_coding\|1/1\|c.863T>C\|p.Leu288Pro\|863/903\|863/903\|288/300\|\|WARNING_TRANSCRIPT_NO_START_CODON | . | 2 dogs of DBVDC homozygous for ALT-allele | |
| 14 | 1388030 | . | T | C | 4015.32 | PASS | AC=5;AF=0.357;AN=14;BaseQRankSum=-4.099;ClippingRankSum=-0.244;DP=293;FS=0.000;MLEAC=5;MLEAF=0.357;MQ=59.93;MQRankSum=0.106;QD=23.48;ReadPosRankSum=-1.341;SOR=0.679;ANN=C\|missense_variant\|MODERATE\|ENSCAFG00000001195\|ENSCAFG00000001195\|transcript\|ENSCAFT00000001847\|protein_coding\|1/1\|c.281A>G\|p.Lys94Arg\|281/936\|281/936\|94/311\|\|,C\|downstream_gene_variant\|MODIFIER\|ELOF1\|ENSCAFG00000028831\|transcript\|ENSCAFT00000047664\|protein_coding\|\|c.*249T>C\|\|\|\|\|3264\|WARNING_TRANSCRIPT_NO_STOP_CODON | . | 48 dogs of DBVDC homozygous for ALT-allele | |
| 14 | 1416639 | . | G | A | 389.74 | PASS | AC=2;AF=0.143;AN=14;BaseQRankSum=0.974;ClippingRankSum=-0.502;DP=96;FS=0.000;MLEAC=2;MLEAF=0.143;MQ=59.66;MQRankSum=-0.143;QD=32.30;ReadPosRankSum=0.788;SOR=0.676;ANN=A\|synonymous_variant\|LOW\|ENSCAFG00000001199\|ENSCAFG00000001199\|transcript\|ENSCAFT00000001850\|protein_coding\|2/3\|c.786C>T\|p.Ala262Ala\|786/1332\|786/1332\|262/443\|\|,A\|downstream_gene_variant\|MODIFIER\|ENSCAFG00000028629\|ENSCAFG00000028629\|transcript\|ENSCAFT00000001863\|protein_coding\|\|c.*939C>T\|\|\|\|\|2734\| | . | synonymous variant | |
| 14 | 1417050 | . | A | G | 1874.38 | PASS | AC=4;AF=0.333;AN=12;BaseQRankSum=-1.219;ClippingRankSum=-1.126;DP=135;FS=2.258;MLEAC=4;MLEAF=0.333;MQ=59.04;MQRankSum=-1.308;QD=24.03;ReadPosRankSum=1.995;SOR=0.904;ANN=G\|missense_variant\|MODERATE\|ENSCAFG00000001199\|ENSCAFG00000001199\|transcript\|ENSCAFT00000001850\|protein_coding\|1/3\|c.377T>C\|p.Leu126Ser\|377/1332\|377/1332\|126/443\|\|,G\|downstream_gene_variant\|MODIFIER\|ENSCAFG00000028629\|ENSCAFG00000028629\|transcript\|ENSCAFT00000001863\|protein_coding\|\|c.*939T>C\|\|\|2323\| | . | 116 dogs of DBVDC homozygous for ALT-allele | |
| 14 | 1571518 | . | T | C | 1529.17 | PASS | AC=4;AF=0.286;AN=14;BaseQRankSum=-0.255;ClippingRankSum=0.581;DP=103;FS=0.000;MLEAC=4;MLEAF=0.286;MQ=60.00;MQRankSum=-0.840;QD=23.17;ReadPosRankSum=-0.248;SOR=0.748;ANN=C\|missense_variant\|MODERATE\|OR2W3\|ENSCAFG00000024736\|transcript\|ENSCAFT00000038187\|protein_coding\|2/2\|c.946A>G\|p.Ile316Val\|946/972\|946/972\|316/323\|\|WARNING_TRANSCRIPT_NO_STOP_CODON | . | 40 dogs of DBVDC homozygous for ALT-allele | |
| 14 | 1695143 | . | T | C | 2050.33 | PASS | AC=5;AF=0.357;AN=14;BaseQRankSum=-0.130;ClippingRankSum=-0.082;DP=159;FS=2.077;MLEAC=5;MLEAF=0.357;MQ=60.00;MQRankSum=0.710;QD=20.50;ReadPosRankSum=0.239;SOR=0.534;ANN=C\|synonymous_variant\|LOW\|FAM71C\|ENSCAFG00000032657\|transcript\|ENSCAFT00000048767\|protein_coding\|4/4\|c.936A>G\|p.Arg312Arg\|1096/1370\|936/969\|312/322\|\| | . | synonymous variant | |
| 14 | 2072958 | . | A | G | 4473.32 | PASS | AC=5;AF=0.357;AN=14;BaseQRankSum=1.651;ClippingRankSum=0.237;DP=341;FS=1.871;MLEAC=5;MLEAF=0.357;MQ=59.95;MQRankSum=-0.604;QD=23.42;ReadPosRankSum=3.444;SOR=0.807;ANN=G\|missense_variant\|MODERATE\|ENSCAFG00000029699\|ENSCAFG00000029699\|transcript\|ENSCAFT00000001934\|protein_coding\|1/1\|c.260A>G\|p.Glu87Gly\|260/939\|260/939\|87/312\|\| | . | 55 dogs of DBVDC homozygous for ALT-allele | |
| 14 | 2181384 | . | C | A | 2735.54 | PASS | AC=6;AF=0.429;AN=14;BaseQRankSum=1.501;ClippingRankSum=-1.223;DP=197;FS=7.509;MLEAC=5;MLEAF=0.357;MQ=60.00;MQRankSum=0.340;QD=22.80;ReadPosRankSum=1.588;SOR=0.393;ANN=A\|missense_variant\|MODERATE\|OR2L13\|ENSCAFG00000032670\|transcript\|ENSCAFT00000001939\|protein_coding\|1/1\|c.146G>T\|p.Arg49Leu\|146/942\|146/942\|49/313\|\|WARNING_TRANSCRIPT_NO_STOP_CODON | . | 45 dogs of DBVDC homozygous for ALT-allele | |
| 14 | 2527459 | . | T | G | 6284.32 | PASS | AC=5;AF=0.357;AN=14;BaseQRankSum=-2.672;ClippingRankSum=0.338;DP=510;FS=1.179;MLEAC=5;MLEAF=0.357;MQ=60.00;MQRankSum=0.225;QD=20.54;ReadPosRankSum=0.446;SOR=0.591;ANN=G\|synonymous_variant\|LOW\|ENSCAFG00000030236\|ENSCAFG00000030236\|transcript\|ENSCAFT00000039095\|protein_coding\|1/2\|c.411T>G\|p.Pro137Pro\|411/960\|411/960\|137/319\|\|WARNING_TRANSCRIPT_NO_STOP_CODON | . | synonymous variant | |
| 14 | 3003654 | . | A | T | 820.69 | PASS | AC=2;AF=0.143;AN=14;BaseQRankSum=1.437;ClippingRankSum=-0.265;DP=103;FS=3.420;MLEAC=2;MLEAF=0.143;MQ=60.00;MQRankSum=-0.442;QD=25.10;ReadPosRankSum=0.872;SOR=1.069;ANN=T\|splice_region_variant&synonymous_variant\|LOW\|AKR1B1\|ENSCAFG00000001290\|transcript\|ENSCAFT00000002000\|protein_coding\|8/10\|c.744A>T\|p.Val248Val\|744/951\|744/951\|248/316\|\|WARNING_TRANSCRIPT_NO_START_CODON,T\|splice_region_variant&synonymous_variant\|LOW\|AKR1B1\|ENSCAFG00000001290\|transcript\|ENSCAFT00000002002\|protein_coding\|8/10\|c.744A>T\|p.Val248Val\|744/951\|744/951\|248/316\|\| | . | synonymous variant | |
| 14 | 3003732 | . | T | C | 625.69 | PASS | AC=2;AF=0.143;AN=14;BaseQRankSum=2.144;ClippingRankSum=-0.227;DP=106;FS=7.598;MLEAC=2;MLEAF=0.143;MQ=60.00;MQRankSum=0.174;QD=29.57;ReadPosRankSum=2.073;SOR=1.093;ANN=C\|synonymous_variant\|LOW\|AKR1B1\|ENSCAFG00000001290\|transcript\|ENSCAFT00000002000\|protein_coding\|8/10\|c.822T>C\|p.Phe274Phe\|822/951\|822/951\|274/316\|\|WARNING_TRANSCRIPT_NO_START_CODON,C\|synonymous_variant\|LOW\|AKR1B1\|ENSCAFG00000001290\|transcript\|ENSCAFT00000002002\|protein_coding\|8/10\|c.822T>C\|p.Phe274Phe\|822/951\|822/951\|274/316\|\| | . | synonymous variant | |
| 14 | 3301140 | . | A | G | 1254.34 | PASS | AC=5;AF=0.357;AN=14;BaseQRankSum=2.109;ClippingRankSum=1.893;DP=105;FS=0.000;MLEAC=4;MLEAF=0.286;MQ=60.00;MQRankSum=-0.461;QD=27.27;ReadPosRankSum=-0.824;SOR=0.616;ANN=G\|missense_variant\|MODERATE\|LRGUK\|ENSCAFG00000001306\|transcript\|ENSCAFT00000046846\|protein_coding\|1/21\|c.154T>C\|p.Phe52Leu\|154/2487\|154/2487\|52/828\|\|,G\|missense_variant\|MODERATE\|LRGUK\|ENSCAFG00000001306\|transcript\|ENSCAFT00000002022\|protein_coding\|1/21\|c.154T>C\|p.Phe52Leu\|154/2484\|154/2484\|52/827\|\|,G\|missense_variant\|MODERATE\|LRGUK\|ENSCAFG00000001306\|transcript\|ENSCAFT00000048140\|protein_coding\|1/20\|c.154T>C\|p.Phe52Leu\|360/2684\|154/2478\|52/825\|\| | . | 27 dogs of DBVDC homozygous for ALT-allele | |

The DNA variants detected by NGS of the enriched exon fragments of the critical region of CFA14 were analyzed with SnpSift and SnpEff (1). The variants were then filtered for homozygosity of the single case and non-homozygosity for the same variant of either of the controls. Variants in SNP clusters, low quality depth or low mapping quality were omitted. The remaining 52 variants are listed. These were further curated on the basis of the integrity of the predicted gene, the predicted effect on the amino acid sequence of the encoded protein, and the presence of the homozygous genotype for the mutation in the WGS database of the Dog Biomedical Variant Database Consortium (*DBVDC*) of 590 dogs from various breeds (2).

| **Supplementary Table S3. Genes in the CFA14 region critical for HNM of Kooiker dogs.** | | | |
| --- | --- | --- | --- |
| start | end | symbol | description |
| 215675 | 220147 | LOC100683343 | brain protein 44-like protein-like |
| 267311 | 300032 | LOC100683414 | transmembrane protein 229A-like |
| 467342 | 473755 | LOC482199 | butyrophilin subfamily 1 member A1-like |
| 502298 | 510286 | LOC482200 | butyrophilin subfamily 1 member A1-like |
| 596292 | 599834 | LOC100683643 | histone H2B type 1-F/J/L-like |
| 603801 | 604199 | LOC482202 | histone H2B type 3-B-like |
| 604505 | 604897 | HIST3H2A | histone cluster 3, H2a |
| 627178 | 627581 | LOC100683710 | histone H3.1-like |
| 634872 | 636181 | LOC100688731 | uncharacterized LOC100688731 |
| 635242 | 639546 | TRIM17 | tripartite motif containing 17 |
| 640441 | 646674 | TRIM11 | tripartite motif containing 11 |
| 664405 | 779354 | OBSCN | obscurin, cytoskeletal calmodulin and titin-interacting RhoGEF |
| 798241 | 805857 | IBA57 | IBA57, iron-sulfur cluster assembly homolog (S. cerevisiae) |
| 811243 | 812545 | GJC2 | gap junction protein, gamma 2, 47kDa |
| 820048 | 826623 | GUK1 | guanylate kinase 1 |
| 826736 | 830107 | MRPL55 | mitochondrial ribosomal protein L55 |
| 832005 | 833538 | C14H1orf35 | chromosome 14 open reading frame, human C1orf35 |
| 834425 | 836418 | LOC100856517 | ADP-ribosylation factor 1-like |
| 864424 | 899698 | WNT3A | wingless-type MMTV integration site family, member 3A |
| 949732 | 956916 | WNT9A | wingless-type MMTV integration site family, member 9A |
| 998161 | 1008129 | PRSS38 | serine protease 44-like |
| 1058896 | 1100724 | SNAP47 | synaptosomal-associated protein, 47kDa |
| 1100847 | 1109978 | JMJD4 | jumonji domain containing 4 |
| 1384518 | 1384769 | LOC100683954 | elongation factor 1 homolog (S. cerevisiae) pseudogene |
| 1387375 | 1388310 | cOR2L17 | cOR2L17 olfactory receptor family 2 subfamily L-like |
| 1416032 | 1417426 | LOC100684658 | suppressor of cytokine signaling 6-like |
| 1582732 | 1601092 | TRIM58 | tripartite motif containing 58 |
| 1689804 | 1699350 | LOC607634 | protein FAM71A-like |
| 1772507 | 1773755 | LOC100856620 | spermine synthase-like |
| 2246473 | 2247455 | LOC482232 | heat shock 70 kDa protein 12A-like |
| 2461699 | 2463259 | LOC100686223 | high affinity copper uptake protein 1-like |
| 2898978 | 2904066 | LOC100687038 | 60 kDa heat shock protein, mitochondrial-like |
| 2991888 | 3006941 | AKR1B1 | aldo-keto reductase family 1, member B1 (aldose reductase) |
| 3120934 | 3149926 | SLC35B4 | solute carrier family 35, member B4 |
| 3193813 | 3301293 | LRGUK | leucine-rich repeats and guanylate kinase domain containing |
| 3312795 | 3327610 | LOC100687273 | 60S ribosomal protein L24-like |
| 3335560 | 4077411 | EXOC4 | exocyst complex component 4 |
| 4229876 | 4507982 | CHCHD3 | coiled-coil-helix-coiled-coil-helix domain containing 3 |

The analysis is based on the NCBI annotation release 104 of CanFam3.1.

**Supplementary References**

1. Cingolani P, Platts A, Wang le L, Coon M, Nguyen T, Wang L, Land SJ, Lu X, Ruden DM. A program for annotating and predicting the effects of single nucleotide polymorphisms, SnpEff: SNPs in the genome of Drosophila melanogaster strain w1118; iso-2; iso-3. Fly (Austin). 2012;6(2):80-92.

2. Jagannathan V, Drogemuller C, Leeb T, Dog Biomedical Variant Database Consortium. A comprehensive biomedical variant catalogue based on whole genome sequences of 582 dogs and eight wolves. Anim Genet. 2019;50(6):695-704.
